# Pediatric Needle Cricothyrotomy: A Case for Simulation in Prehospital Medicine

**DOI:** 10.15766/mep_2374-8265.10589

**Published:** 2017-06-02

**Authors:** Jason P. Stopyra, Jamie L. Wright, Michael T. Fitch, Michael S. Mitchell

**Affiliations:** 1 Assistant Professor, Departments of Emergency Medicine and Pediatric Emergency Medicine, Wake Forest School of Medicine of Wake Forest Baptist Medical Center; 2 Resident Physician, Departments of Emergency Medicine and Pediatric Emergency Medicine, Wake Forest School of Medicine of Wake Forest Baptist Medical Center; 3 Professor, Departments of Emergency Medicine and Pediatric Emergency Medicine, Wake Forest School of Medicine of Wake Forest Baptist Medical Center

**Keywords:** Simulation, Anaphylaxis, Emergency Medical Services, Pediatrics, Prehospital Medicine, Needle Cricothyrotomy, Airways

## Abstract

**Introduction:**

A patient that cannot be oxygenated or ventilated requires immediate and effective assessment, treatment, and transportation. Pediatric needle cricothyrotomy is used infrequently, therefore providers have a tendency to lose proficiency. Simulation training and evaluation are valuable tools to improve provider experience and skill.

**Methods:**

A case was designed involving a 3-year-old male with a peanut allergy that presents with rash, swelling, and severe respiratory distress. The patient's respiratory distress and swelling worsens despite treatment with epinephrine and other allergic reaction medications. The patient then becomes unresponsive and impossible to oxygenate or ventilate. The primary objective of this case is airway management with needle cricothyrotomy in the pediatric population. A secondary objective is appropriate postprocedure management including appropriate ventilation rates and emergency medical transportation methods.

**Results:**

This case was initially presented to 45 paramedics. Provider comfort with managing airway emergencies in young children improved from 47% to 89%. Confidence in performing pediatric needle cricothyrotomy improved from 16% to 87%. All providers felt the exercise was valuable and 98% felt the simulation provided appropriate realism.

**Discussion:**

This scenario provides an outstanding opportunity for paramedic evaluation and training in pediatric needle cricothyrotomy and significantly improved the comfort level of providers' management of a failed pediatric airway. As we reflected on the use of this module, it was apparent that this was a very beneficial opportunity to spend one-on-one time between participants and their medical director. The training staff also benefited from the repeated emphasis of good assessment and treatment of a complex patient scenario.

## Educational Objectives

By the end of this module, learners will be able to:

1. Identify an apneic patient without a patent airway.
2. Demonstrate the appropriate initial approach of airway management.
3. Identify indications for needle cricothyrotomy.
4. Select the proper equipment for needle cricothyrotomy.
5. Demonstrate proper patient positioning and identification of anatomical landmarks.
6. Demonstrate appropriate technique in placing a needle cricothyrotomy airway.
7. Ensure proper placement of the airway with carbon dioxide detectors and oxygen monitors.
8. Identify the appropriate ventilation rate with a bag valve mask.
9. Determine transportation method to an appropriate facility.

## Introduction

Patients with inadequate oxygenation are commonly encountered by prehospital providers. The first few minutes of care provided to these patients can be chaotic. The stress that a prehospital provider experiences while taking care of a hypoxic patient in respiratory distress can be further elevated when the patient is a child. Educational resources are needed to help train providers so that they can make appropriate and methodical clinical decisions early in patient encounters. A patient that a provider cannot oxygenate or ventilate is critically ill and requires immediate and effective assessment, treatment, and transportation.

Advanced airway procedures are necessary in the emergency department and the prehospital setting. These procedures have significant risk but may be the only option to providers in order to avoid the impending demise of a critically ill patient. Pediatric needle cricothyrotomy is used infrequently and therefore providers have a tendency to lose proficiency.^1–3^ A review of statewide data from the North Carolina Emergency Medical Services Performance Improvement Center revealed that a pediatric needle cricothyrotomy was attempted seven times between 2011–2014. Simulation training and evaluation are valuable tools to improve provider experience and skill, and the current educational materials are designed to improve familiarity and comfort with this uncommon procedure.

This case of a pediatric patient that requires a definitive airway provides an opportunity for providers to assess and manage an unstable pediatric patient. Pediatric needle cricothyrotomy is a clinical skill available to prehospital agencies that requires careful consideration of multiple factors to safely manage airway emergencies. Needle cricothyrotomy remains the safest alternative in the “can't intubate, can't ventilate” scenario most commonly found in upper airway obstruction.^4,5^ All attempts at securing the airway should have been attempted and failed (bag valve mask, endotracheal tube, alternate airway) prior to considering airway management with needle cricothyrotomy. This airway intervention is only a temporary solution and should not be used for longer than 45 minutes.^6,7^ If ground transportation time is longer than this time period, air transportation may be necessary.

Previously published resources have focused on in-hospital resident training both in the emergency department and operating room. This novel resource provides the background, instructions, and material list for an out-of-hospital simulation case or oral board exam appropriate for paramedic students, emergency medical technicians-paramedics, and critical-care transport providers, such as paramedics and nurses.

## Methods

This educational module is centered on a pediatric “can't intubate, can't ventilate” scenario in the prehospital setting. It can be utilized by emergency medical services (EMS) training departments or medical directors to provide continuing education and an objective way to evaluate a provider's ability to negotiate this type of critical care patient encounter. The case itself is presented in the simulation case file (Appendix A) that includes information needed to implement this case. A PowerPoint presentation (Appendix B) suitable for use before or after participants have completed the case is available, along with a participant evaluation tool (Appendix C) and a pre- and posttest (Appendix D).

This case can be implemented using equipment such as a Laerdal SimJunior, Life/form Complete Child CRiSis Manikin, or another appropriate high-fidelity patient simulator. Alternatively, with a few modifications the case can also be completed using low-fidelity patient mannequins or even used in an oral board presentation format. To fully implement this teaching module, all formats will require a separate skill completion component. This can be performed on either on a cadaveric fetal pig (Appendix E) or an artificial low-cost “hardware store” model (Appendix F). An additional document explaining the correct procedures for airway management and needle cricothyrotomy (Appendix G) is also included, as are files containing visual stimuli (Appendix H–J). This case can be presented with a single evaluator providing case information and role-playing various aspects of the case. The case may be enhanced with additional actors serving as detailed in the case file.

## Results

This scenario has been presented to 45 paramedics over two different days to evaluate their ability to maintain competence in pediatric needle cricothyrotomy. The scenario was used as a stand-alone training tool to fulfill a State Office of EMS mandate to complete yearly training in pediatric needle cricothyrotomy for paramedics under medical direction of one of the authors.

Our experience to date has been that experienced paramedics are able to successfully negotiate this case without significant difficulty. As we have stressed the importance of capnography and airway adjuncts usage, the paramedics have been more successful in the management of these type of patients.

Some participants have failed to properly recognize the need for an airway early in the case. Due to the emergent nature of the airway compromise in this case, it cannot be successfully completed without identifying and successfully managing the airway. Similarly, participants who did not initiate adequate initial airway management had difficulty completing the case successfully, as immediate airway maneuvers and utilization of appropriate airway adjuncts are vital in oxygenating the patient. Participants that recognized the need for a definitive airway but either did not attempt a blind insertion of an airway device or a direct laryngoscopy endotracheal insertion did not appropriately assess the airway in a step-wise manner. Protocols are in place that improve patient outcomes and decrease risks when attempting a definitive airway prior to attempting a surgical airway.

Three specific areas are highlighted as impeding a successful needle cricothyrotomy: inappropriate placement of the needle cricothyrotomy, failure to use capnography, and failure to secure the catheter in place. Knowledge of anatomical landmarks, criteria for need for placement of a needle cricothyrotomy, and proficiency in the procedural steps are vital in the successful placement of an airway in this patient population. It is now standard practice to confirm definitive airway placement with capnography in the prehospital setting. Providers must be vigilant for changes in patient condition, as this may indicate that the airway is not correctly placed due to the provider's inability to secure it in place.

A survey was completed by each of the 45 paramedics who completed the scenario. Provider comfort in successfully managing airway emergencies in young children improved from 47% to 89%. Confidence in performing pediatric needle cricothyrotomy improved from 16% to 87%. All providers felt the exercise was valuable and 98% felt the simulation model used provided appropriate realism to learn the procedure.

The participant evaluation tool provides a guide for the evaluator to move through the case and record the provider's actions in real time. We consistently use a threshold of 75% as the required value to pass the scenario. Additional penalties are assessed when participants failed to perform certain tasks highlighted in red. Please note that there is an important line toward the bottom of scenario file that allows the instructor or evaluator to fail the participant despite their total score. This is in place for the rare occasion where a provider causes the patient harm. In the event that a provider does not pass, we suggest the training staff or medical director provide focused, immediate remediation. If there are still gaps in the provider's knowledge they should engage in guided self-study. It is at the discretion of the training staff or medical director whether to offer another opportunity to attempt to pass this or a similar case at a later time.

## Discussion

This scenario provides an outstanding opportunity for paramedic evaluation and training in pediatric needle cricothyrotomy. The institutional training department and medical director can use this case to teach the critical processes of needle cricothyrotomy. As we reflect on the use of this module, it was apparent that this is a very beneficial opportunity for both participants and medical directors to spend one-on-one time with each other. The training staff also benefits greatly from the repeated emphasis of good assessment and treatment of a complex patient scenario.

Over time, the scenario and evaluation of the participant has gone through several versions. EMS professionals focus on their environment and surroundings. As a result, several environmental descriptors have been added to assist the evaluator who may not be as familiar with the prehospital environment. At the same time, we learned that participants do not collect all the information they should from a rapid primary survey. Providers need to know how to vocalize their assessment, as well as perform it when asked to explain their actions and treatment decisions.

The models used to allow providers to perform needle cricothyrotomy were a focus of many modifications. Early on we found that the fetal pigs had characteristics that made them necessary, but that a durable model was required to allow providers the opportunity to practice sufficiently to develop muscle memory over time. This more durable model was developed using supplies available at a local hardware store combined with an IV tourniquet. We also noted that practice during the exercise and other times made the fetal pig model much more useful. The providers were more focused on anatomy and less on figuring out how to perform the procedure.

Patient positioning to palpate the cricothyroid membrane on actors was very useful. The use of the stretcher was an excellent tool to assist with this. Providers were encouraged to ask family and friends permission to identify their cricothyroid membrane.

During module preparation, it became quickly apparent that the fetal pig model required some minimal dissection to the larynx and trachea to allow providers to feel the anatomy. It was impossible to feel from the skin surface. In response to learner feedback, the fetal pig model was prepped further prior to use. It was determined that the airways required suctioning and air insufflation prior to provider use. This preparation made the model very useful and similar to the anatomy present in a small, 12-month-old child.

The framework used here has been adopted by the services that helped develop it to be used annually in order to reinforce and prove competency in emergent pediatric airway management. It is also gained the audience of the local paramedic training programs contained within community colleges as a valuable resource to teach this subject.

This model requires a considerable amount of preparation prior to use. Whether the hardware store or fetal pig model are used, both require preplanning. In addition, multiple stations and therefore multiple trainers and evaluators were necessary to allow multiple providers to complete the module in a day. The module took approximately 90 minutes for providers to complete.

Even when a high-fidelity simulator was used, it was necessary for the skill to be performed on an alternate model. This took away from the realism of the module. Finally, the result that determined the success of this module was based solely on the trainees' perceived comfort with the procedure.

## Appendices

A. Simulation Case.docx
B. PowerPoint Presentation.pptx
C. Participant Evaluation Tool.docx
D. Pre- and Posttest.docx
E. Fetal Pig Model.docx
F. Hardware Store Model.docx
G. Correct Procedure Technique Explained.docx
H. Needle Kit Image.JPG
I. Angioedema Image.JPG
J. Urticaria Image.jpg All appendices are peer reviewed as integral parts of the Original Publication.
