# Supplementary material for: Pediatric Needle Cricothyrotomy: A Case for Simulation in Prehospital Medicine
Source: MedEdPORTAL. 2017 Jun 2;13:10589. doi: 10.15766/mep_2374-8265.10589 (PMC6338176; doi:10.15766/mep_2374-8265.10589)
Supplement: Supplementary file 1 — A. Simulation Case.docx B. PowerPoint Presentation.pptx C. Participant Evaluation Tool.docx D. Pre- and Posttest.docx E. Fetal Pig Model.docx F. Hardware Store Model.docx G. Correct Procedure Technique Explained.docx H. Needle Kit Image.JPG I. Angioedema Image.JPG J. Urticaria Image.jpg [file mep-13-10589-s001.zip › A. Simulation Case.docx]

| **Appendix A: MedEdPORTAL Simulation Case**    **SIMULATION CASE TITLE:  Pediatric Needle Cricothyrotomy: A Case for Simulation in Prehospital Medicine**    **AUTHORS: Stopyra JP, Wright JL, Fitch MT, Mitchell MS** | |
| --- | --- |
| **PATIENT NAME:  Billy**    **PATIENT AGE: 3yrs**    **CHIEF COMPLAINT: Allergic reaction** | |
|  | |
| **Brief narrative description of case** | Three-year-old male with peanut allergy complaining of rash and difficulty breathing.    The overall goal for this case is to provide a platform for the evaluation of a prehospital provider’s ability to properly perform pediatric needle cricothyrotomy. |
| **Primary Learning Objectives** | 1. Identify an apneic patient without a patent airway. 2. Demonstrate the appropriate initial approach of airway management. 3. Identify indications for needle cricothyrotomy. 4. Select the proper equipment for needle cricothyrotomy. 5. Demonstrate proper patient positioning and identification of anatomical landmarks. 6. Demonstrate appropriate technique in placing a needle cricothyrotomy airway. 7. Ensure proper placement of the airway with carbon dioxide detectors and oxygen monitors**.** |
| **Critical Actions** | 1. Rapid administration of Epinephrine 0.15mg IM (1:1000) 2. Start an intraosseous line using appropriate technique in a timely fashion. 3. Perform a needle cricothyotomy |
| **Learner Preparation** | This is a simulated case that will require you to assess and treat a simulated patient. If you feel the need to perform a procedure, state the procedure you wish to perform and the evaluator will direct you to the proper simulator. |

| **Initial Presentation** | | | |
| --- | --- | --- | --- |
| **EMS Dispatch** | Three-year-old male Allergic Reaction – Rash and Respiratory Distress  Responding to a residence. | | |
| **Environment** | 8pm. No weather or temperature issues. | | |
| **Initial vital signs** | Temp: 98.6F (37C) BP: 118/78 HR: 142 RR:40 Pulse Ox: 92% EtCO2: 30 | | |
| **Overall Appearance** | 3-year-old male kneeling on the floor in tripod position in severe distress wearing pajamas. Patient’s face appears red and swollen. No abnormal breath sounds are heard from across the room. | | |
| **Actors and roles in the room at case start** | A crew member should be present to assist the lead provider. It would be advisable to have this person trained as a paramedic or nurse. They should not perform any critical assessment, initiate any treatment or perform any procedure critical to the successful completion of this case. It is acceptable for example to have them attempt IV/IO access, give medications at the direction of lead provider, attach monitoring equipment and obtain vital signs.  The patient’s grandmother is present. She is appropriately concerned about her grandson. Medical training is not required to play her role. She will tell the crew the patient’s HPI, PMH, Meds, Allergies and family history. | | |
| **Primary Survey** | Level of Consciousness: Awake, alert in severe distress  Airway: Angioedema to lips/tongue  Breathing: Stridor and wheezing present with minimal air movement  Circulation: Tachycardic with capillary refill in distal extremities of 3-4 seconds  Disability: Moves all extremities, pupils reactive to light and equal  Exposure: Diffuse urticarial rash, no trauma, toxins/exposures appreciated | | |
| **HPI** | Grandmother states: “Billy was watching TV when he started itching. Before I knew it he had hives all over and started to struggle to breath. I gave him his Epi Pen and called 911. He doesn’t seem to be improving.”    Last meal – 3 hours ago | | |
| **Past Medical/Surgical History** | **Medications** | **Allergies** | **Family History** |
| Allergic to peanuts | Epi-Pen Jr | NKDA | Hypertension, Diabetes |
| **Physical Examination** | | | |
| **General** | 15kg normally developed 3 year old male in severe distress | | |
| **HEENT** | Angioedema to eyes (Stimulus 1) lips & tongue. Atraumatic. | | |
| **Neck** | No JVD or tracheal deviation | | |
| **Lungs** | Rapid rate with poor air movement. Stridor and wheezing are audible and heard upon auscultation. Intercostal retractions and accessory muscle use are appreciated. | | |
| **Cardiovascular** | Tachycardic without murmurs, rubs, gallops | | |
| **Abdomen** | Non-tender, no hepatomegaly | | |
| **Neurological** | Awake, alert, moving all extremities. | | |
| **Skin** | Diffuse urticaria (Stimulus 2) | | |
| **Musculoskeletal** | Normal tone, atraumatic, no deformity | | |
| **GU** | Normal circumcised male genitalia. | | |

| **Instructor Notes - Changes and CASE Branch Points** | | |
| --- | --- | --- |
| **Intervention / Time point** | **Change in Case** | **Additional Information** |
| *Epinephrine is given by intramuscular injection for suspected anaphylaxis* | *Patient heart rate increases by 20 beats per minute over next 1 minute.  Respiratory distress and swelling continues to worsen.* |  |
| 5 minutes after arrival | Patient becomes somnolent. Minimally responsive to painful stimuli. | Vitals: Temp: 98.6F (37C) BP: 84/54 HR: 152 RR:20 Pulse Ox: 82% (w/ O2) EtCO2: 65. Mannequin should become cyanotic if possible. |
| Parenteral access | No peripheral access possible. Intraosseous access achieved on first attempt. | Fluid bolus, diphenhydramine, steroid, and Albuterol may be administered, but will not change patient’s condition. |
| Repeat Epinephrine given 5 minutes after first dose | Continued worsening of swelling. Patient becomes unresponsive. | Provider moves to administration of IV/IO Epi. Contact with Medical Control may be required by local protocols. No improvement in patient’s condition. |
| Airway management  (Appendix E) | Unsuccessful in all oro/nasopharyngeal approaches |  |
| Performs needle cricothyrotomy  (Appendix E) | Oxygenation improves | Vitals: Temp: 98.6F (37C) BP: 98/62 HR: 138 RR:40 Pulse Ox: 90% EtCO2: 54 |
| Initiates Rapid Transport | Continuous monitoring |  |

1. **Lab set-up** - Depending on available resources, this case may be set-up as in a residence, a training room or simulation lab with the pediatric mannequin positioned seated in a chair.
2. **Mannequin Set-up** - The mannequin will be a clothed, pediatric (< 12 year old) sized mannequin. At the beginning of the simulation, there are no monitor leads on the patient, no IV access has been obtained, and the patient is not on oxygen. Moulage can be applied to the mannequin to reveal erythematous, urticarial rash. Angioedema will likely have to be verbalized.
3. **Props** - Available for use will be a portable heart monitor/defibrillator with leads, blood pressure cuff, pulse oximeter, and end tidal CO2 monitor. Supplemental oxygen by nasal cannula, face mask, and bag valve mask should be available in pediatric sizes. Additional advanced airway tools available include oral and nasal pharyngeal airways, blind insertion airway devices, endotracheal tubes and direct laryngoscopy devices (handles and blades), and bougie. The supplies for needle cricothyrotomy placement will be available including an 18 gauge or larger angiocath, saline half-filled syringe, size 3 endotracheal tube adapter, and alcohol swab. (See Appendix F)
4. **Audiovisual** – Stimuli 1 & 2
5. **Ideal Scenario Flow** *-* The providers enter the room to find a pediatric patient in severe respiratory distress. They immediately perform a primary survey, obtain vital signs, administer supplemental oxygen and IM epinephrine. Airway adjuncts (OPA/NPA) are not able to be inserted, but when patient’s mental status declines attempts to ventilate with BVM should be attempted. They assess the patient for IV access, but when told this is impossible they quickly move to and achieve intraosseous access. Repeat epinephrine, fluid bolus, diphenhydramine, H2 blocker, corticosteroids, and albuterol may be administered. Due to the severity of this patient’s presentation the learner should recognize that these treatments will likely be unsuccessful and an Epinephrine drip will be necessary. Due to the progressive swelling in the patient’s airway and an inability to secure it in the usual fashion, the learner should turn their attention to performing needle cricothyrotomy.(Appendix E) This skill should be performed on the cadaveric fetal pig (Appendix B1) or “hardware store” model. (Appendix B2) After this is successfully performed the learner should efficiently transport the patient to an appropriate Emergency Department. During transport a history can be obtained from the grandmother. A complete physical exam can also be performed which will reveal improving urticarial and swelling if intravenous epinephrine has been administered.
6. **Anticipated Management Mistakes** -
7. Failure to progress quickly to IO access
8. Failure to quickly recognize the need for airway management.
9. Failure to position patient in best position to successfully identify cricothyroid membrane.
10. Uncertainty about indications and contraindications for needle cricothyrotomy
11. Lack of familiarity in performing needle cricothyrotomy
12. Failure to adequately secure the catheter in place
13. Failure to use capnography.
14. Failure to progress to Epinephrine drip.
15. Failure to recognize the need to transfer the patient to the most appropriate facility by the most appropriate method.
16. **Debriefing Plan**
    1. **Method of Debriefing** – A six element Debriefing Assessment for Simulation in Healthcare (DASH) format is recommended.^1,2^
       1. Create engaging learning environment. Clarify module objectives and expectations in a comfortable environment with a commitment to respecting learner and their perspective
       2. Maintain an engaging context for learning. Respectfully assist learner in coping with a limited realism situation while setting tone regarding realism.
       3. Structure the debriefing in an organized way. Begin by encouraging participant to share their initial reaction to the simulation. Then guide analysis of the participant to make sense of the simulation events. Finally summarize learning from the session.
       4. Provoke interesting and engaging discussions to foster reflective practice.
       5. Identify and explore performance gaps. See Appendix C (may need to be adjusted per local practice/protocol). Ensure the following knowledge:
          1. Indications for a pediatric needle cricothyrotomy
          2. This is a procedure only to be used in the gravest of situations
          3. Predictors of a difficult needle cricothyrotomy
          4. The optimal position of a patient for needle cricothyrotomy
          5. Major landmarks of the neck that need to be identified
          6. Correct needle cricothyrotomy technique
          7. Appropriate ventilation rate after needle cricothyrotomy
       6. Help trainee achieve or sustain good future performance. Assure that all performance gaps are filled through discussion and teaching to demonstrate a firm grasp of the subject and objective fulfilment.
17. **Reference:**
18. Simon R, Rudolph JW, Raemer DB. Debriefing Assessment for Simulation in Healthcare. Cambridge, MA; 2009. Available at:<http://www.harvardmedsim.org/debriefing-assesment-simulation-healthcare.php>
19. Brett-Fleegler M1, Rudolph J, Eppich W, Monuteaux M, Fleegler E, Cheng A, Simon R. Debriefing assessment for simulation in healthcare: development and psychometric properties. Simul Healthc. 2012 Oct;7(5):288-94.
