# Supplementary material for: Pediatric Needle Cricothyrotomy: A Case for Simulation in Prehospital Medicine
Source: MedEdPORTAL. 2017 Jun 2;13:10589. doi: 10.15766/mep_2374-8265.10589 (PMC6338176; doi:10.15766/mep_2374-8265.10589)
Supplement: Supplementary file 1 — A. Simulation Case.docx B. PowerPoint Presentation.pptx C. Participant Evaluation Tool.docx D. Pre- and Posttest.docx E. Fetal Pig Model.docx F. Hardware Store Model.docx G. Correct Procedure Technique Explained.docx H. Needle Kit Image.JPG I. Angioedema Image.JPG J. Urticaria Image.jpg [file mep-13-10589-s001.zip › B. PowerPoint Presentation.pptx]

## Slide 1
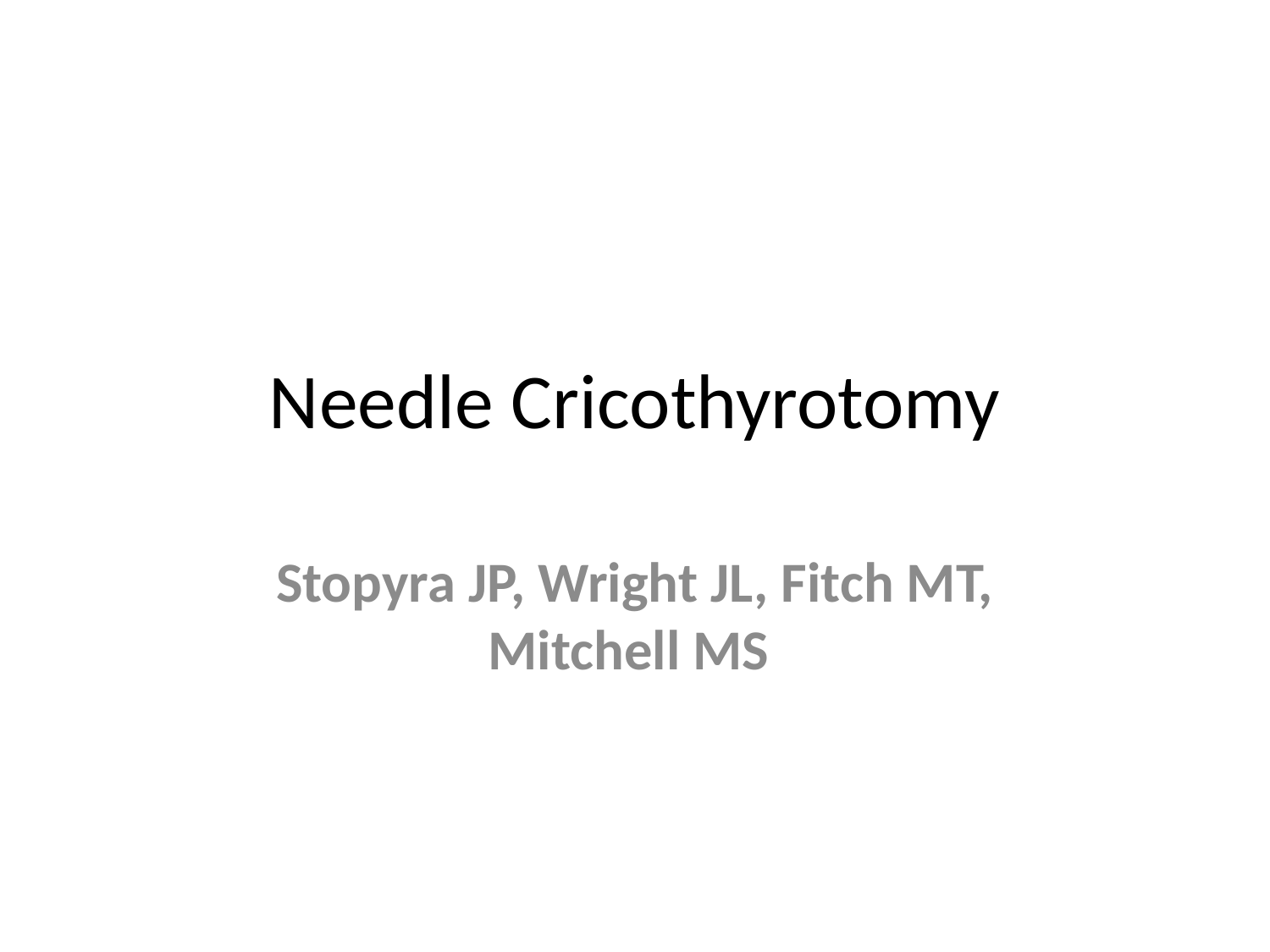

# Needle Cricothyrotomy
Stopyra JP, Wright JL, Fitch MT, Mitchell MS

## Slide 2
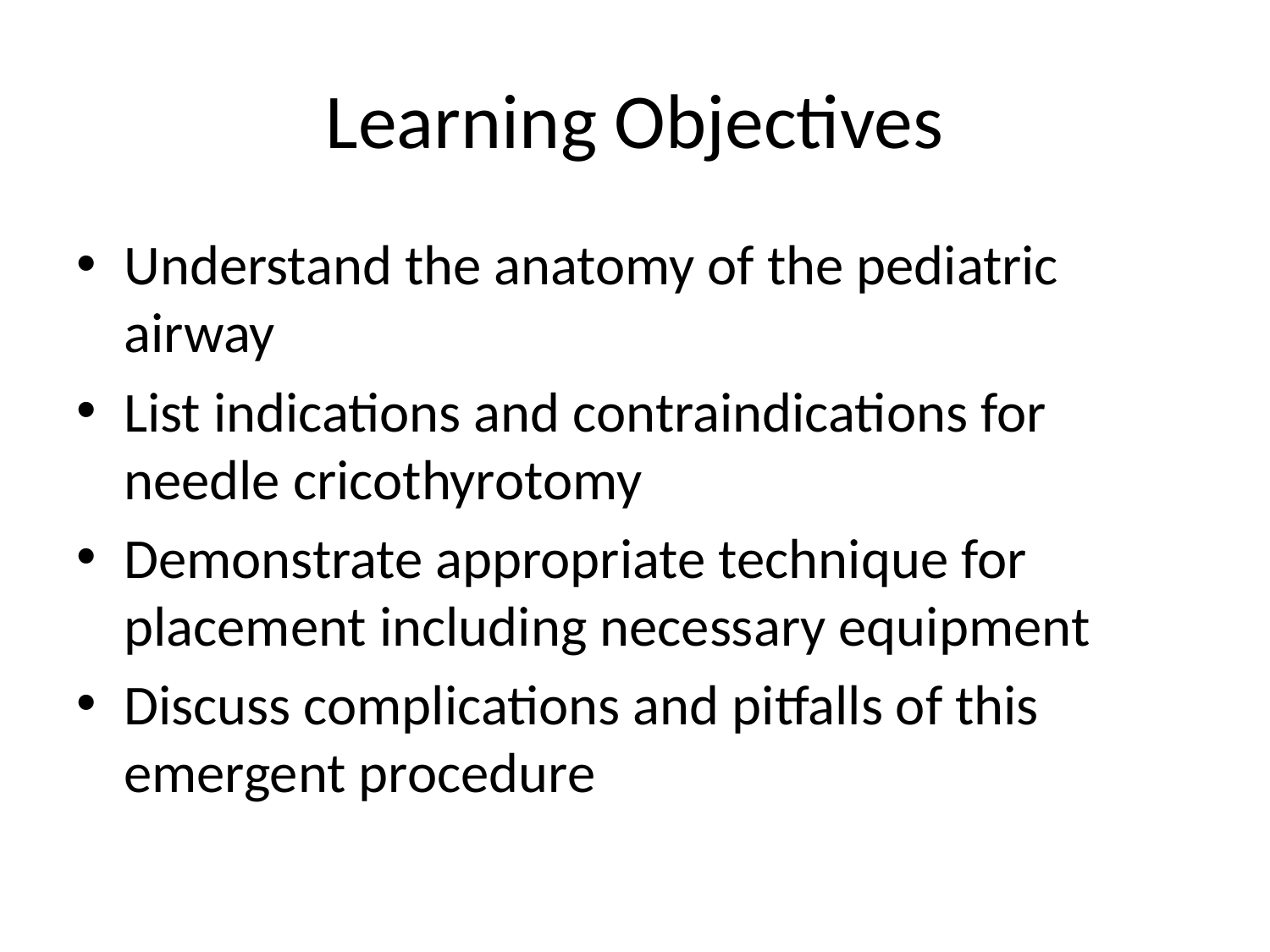

# Learning Objectives
Understand the anatomy of the pediatric airway
List indications and contraindications for needle cricothyrotomy
Demonstrate appropriate technique for placement including necessary equipment
Discuss complications and pitfalls of this emergent procedure

## Slide 3
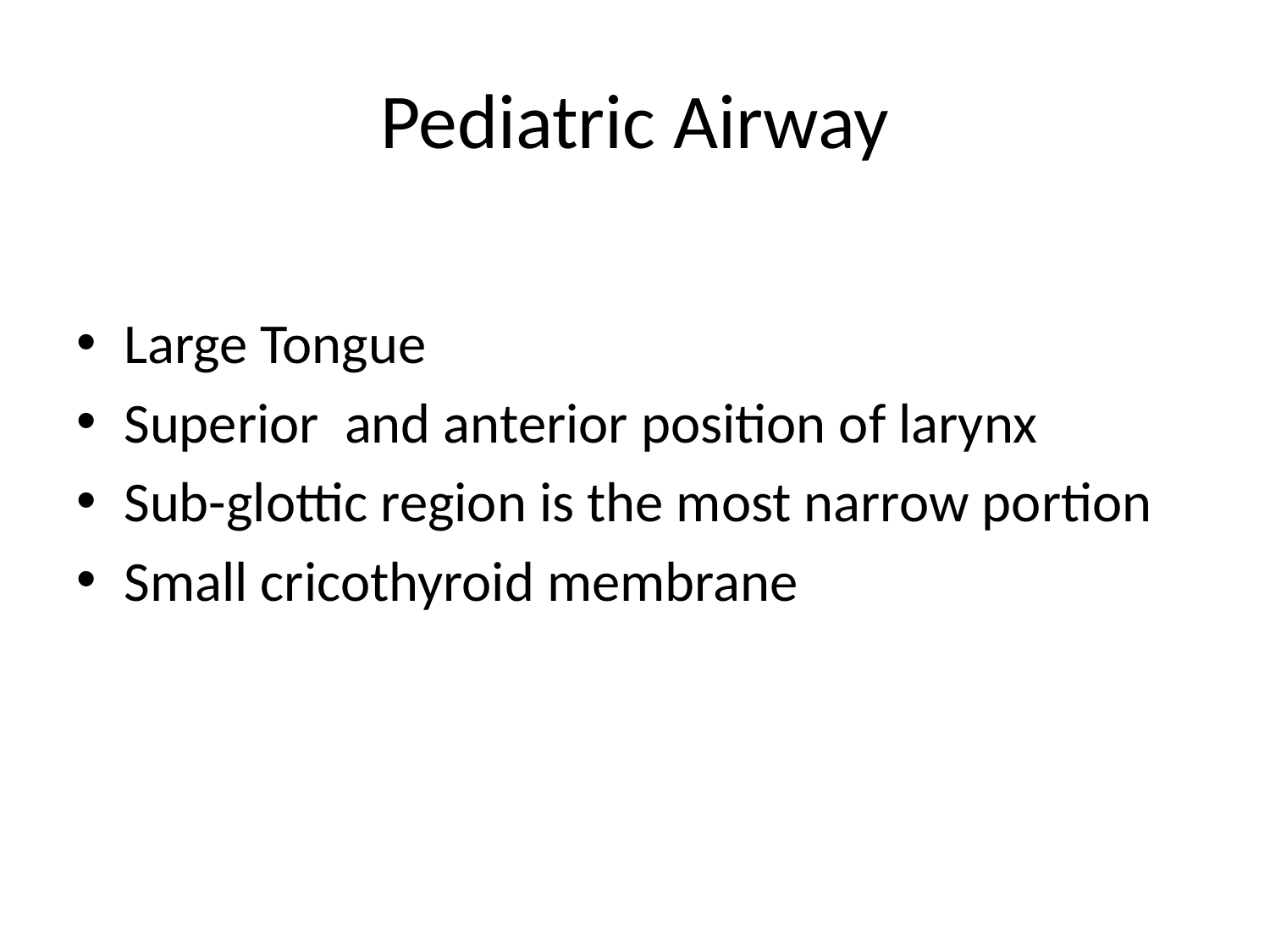

# Pediatric Airway
Large Tongue
Superior and anterior position of larynx
Sub-glottic region is the most narrow portion
Small cricothyroid membrane

## Slide 4
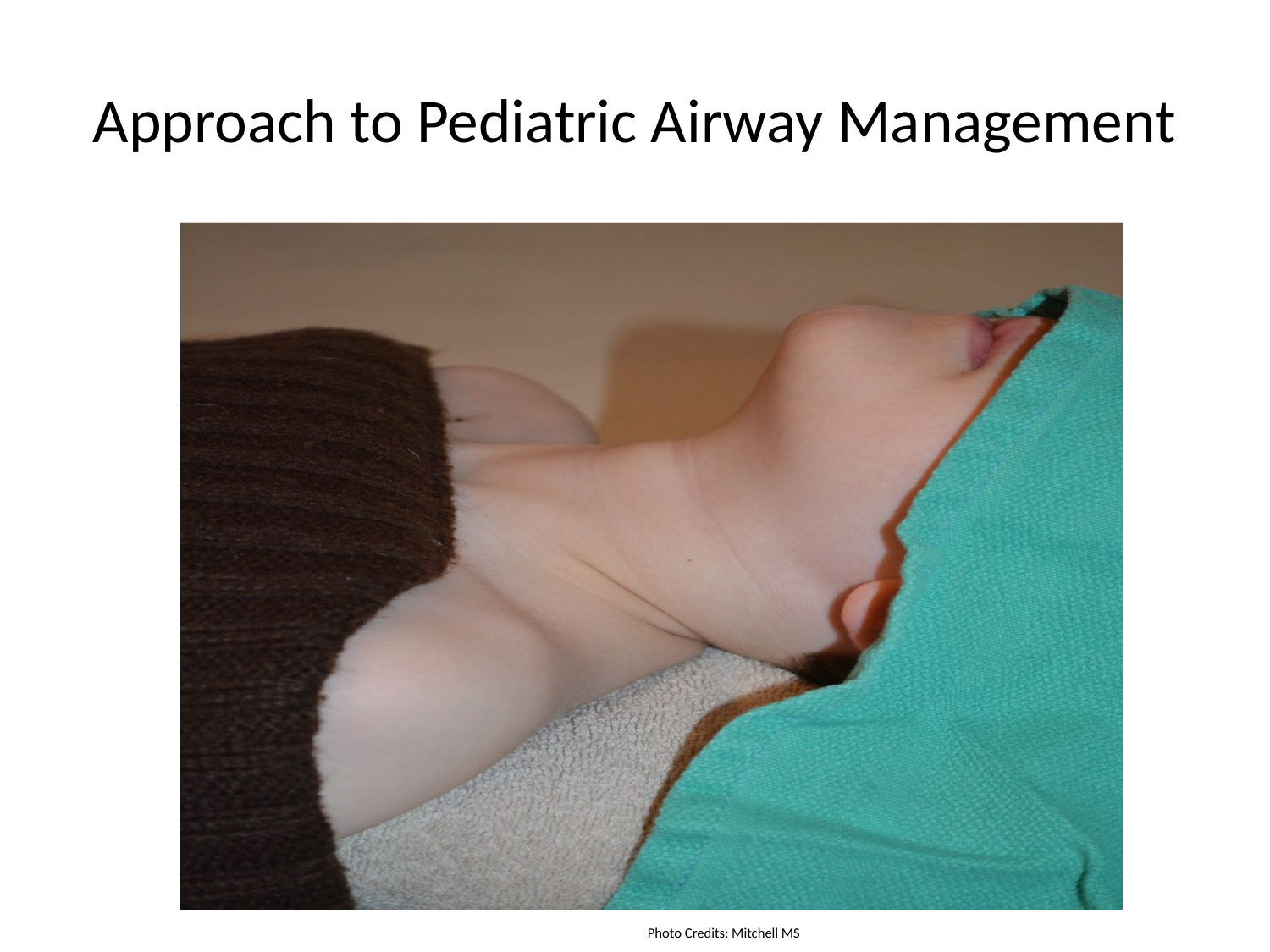

# Approach to Pediatric Airway Management
Photo Credits: Mitchell MS

## Slide 5
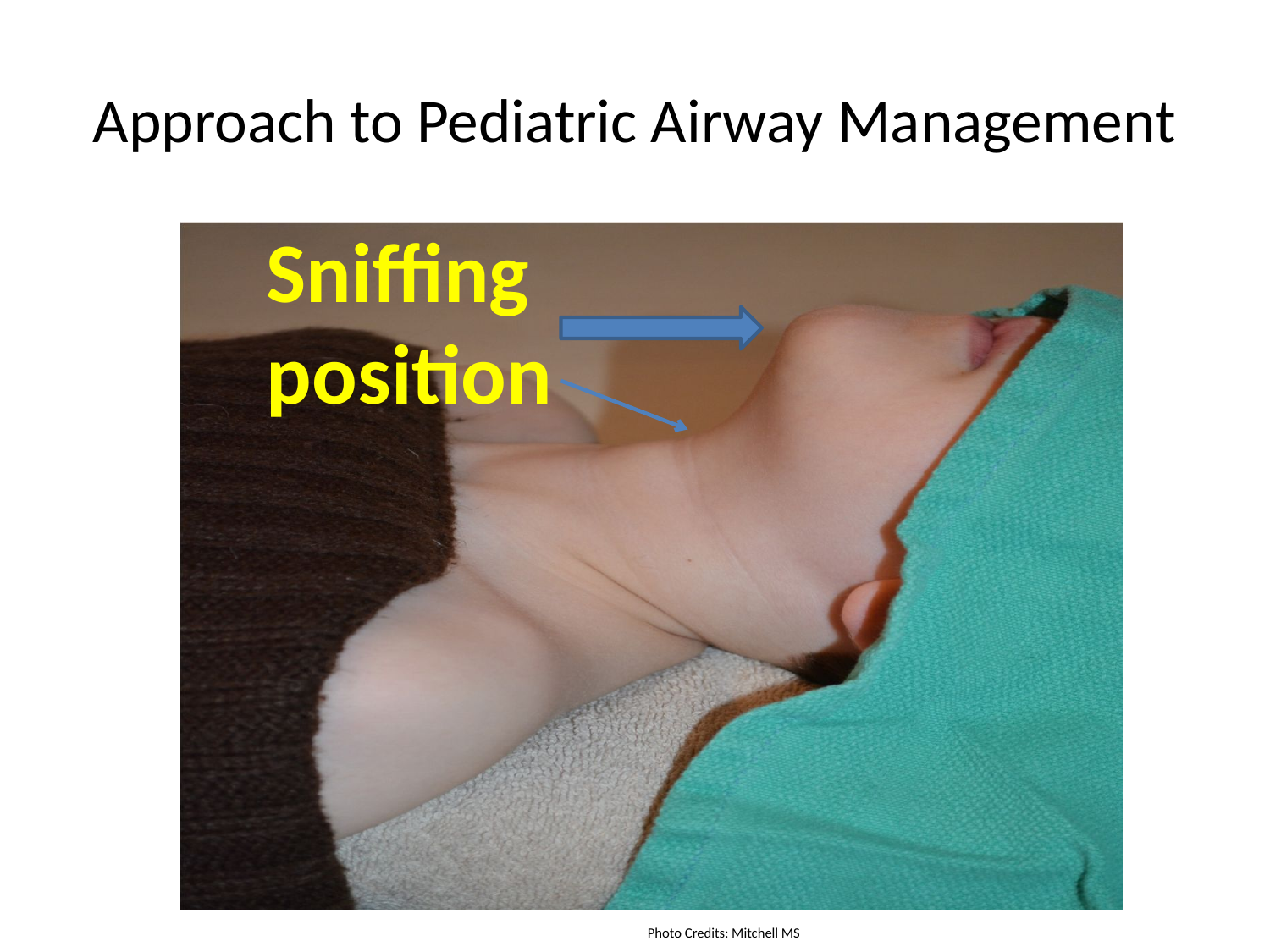

# Approach to Pediatric Airway Management
Sniffing position
Photo Credits: Mitchell MS

## Slide 6
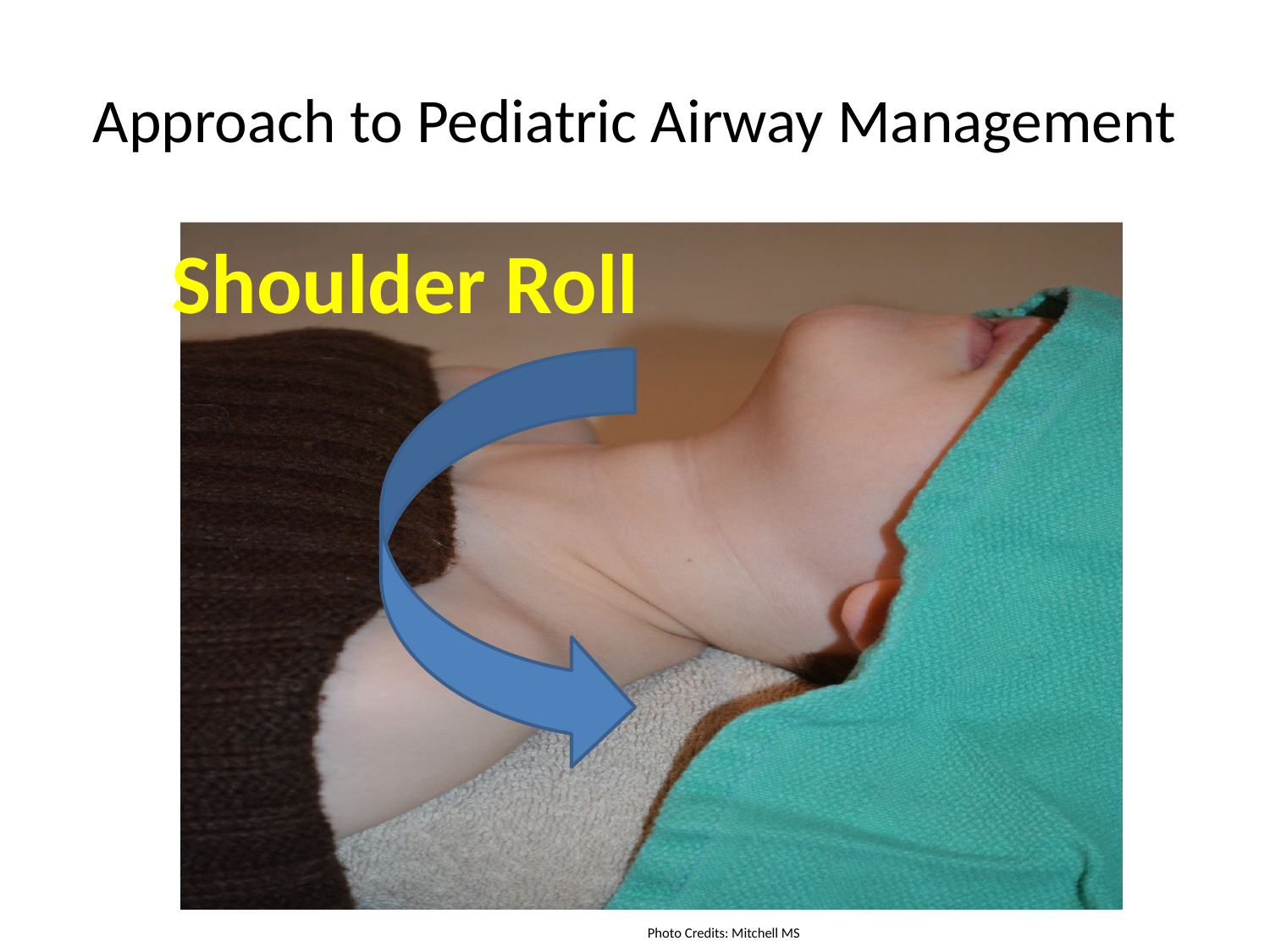

# Approach to Pediatric Airway Management
Shoulder Roll
Photo Credits: Mitchell MS

## Slide 7
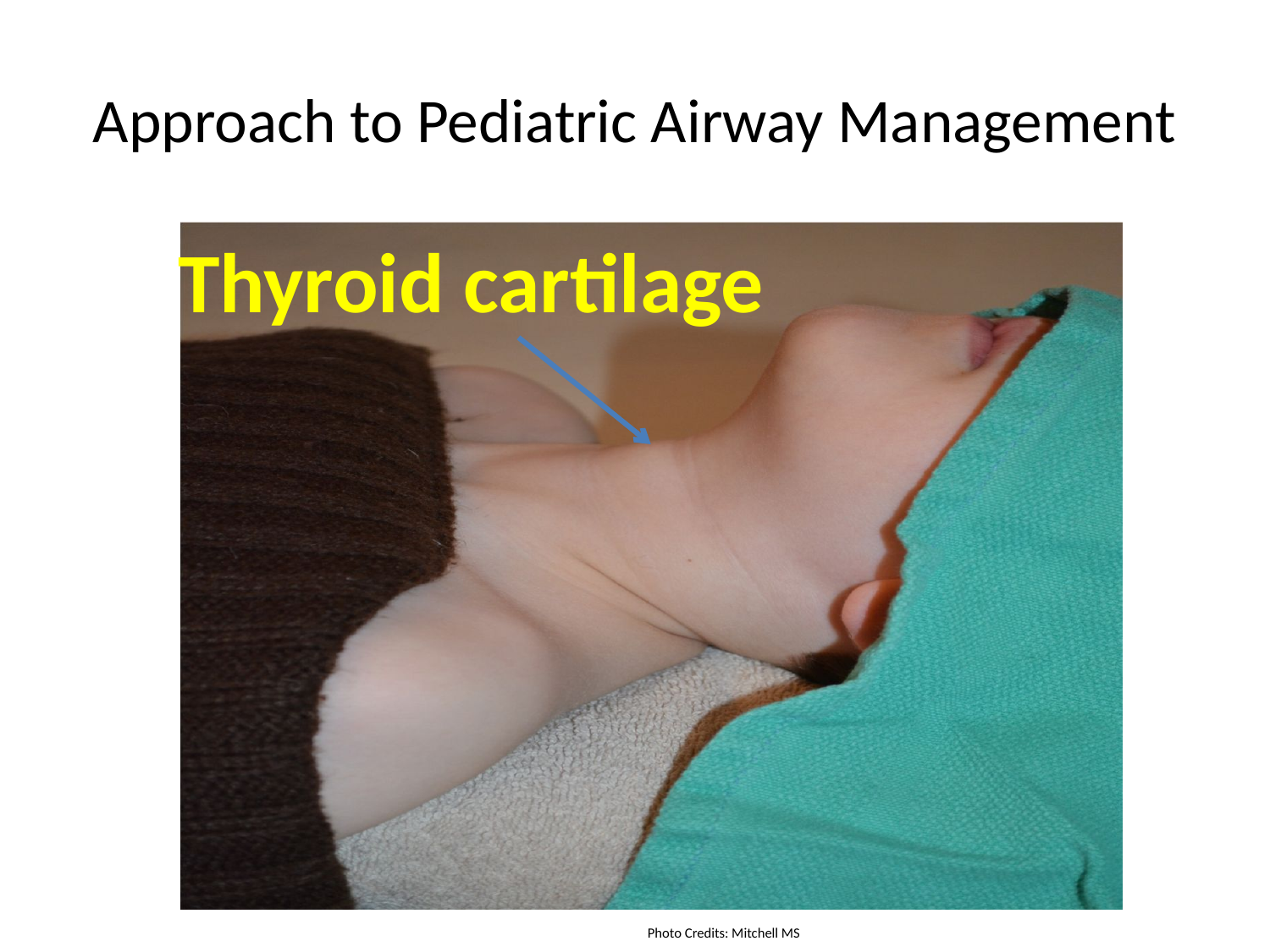

# Approach to Pediatric Airway Management
Thyroid cartilage
Photo Credits: Mitchell MS

## Slide 8
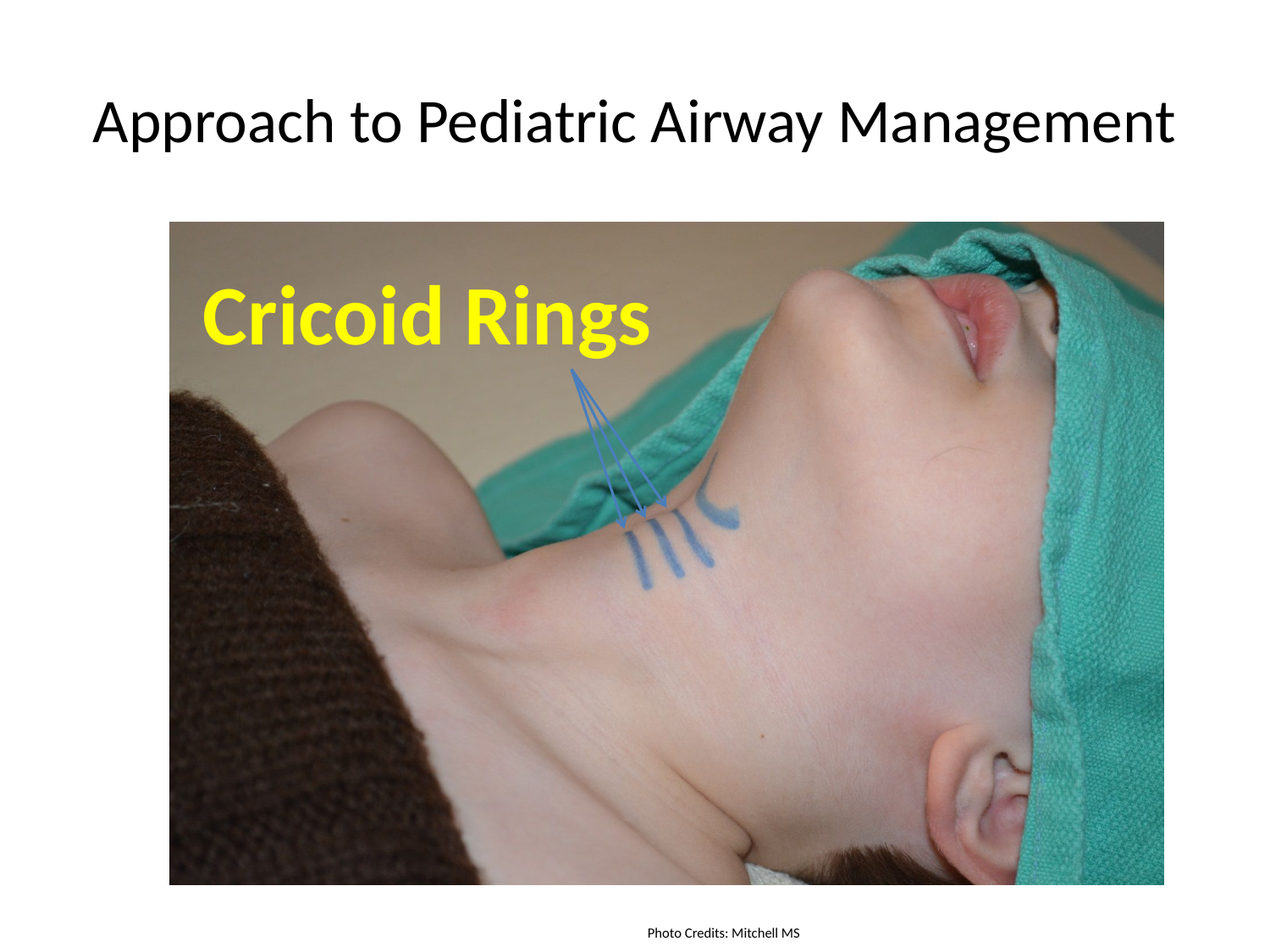

# Approach to Pediatric Airway Management
Cricoid Rings
Photo Credits: Mitchell MS

## Slide 9
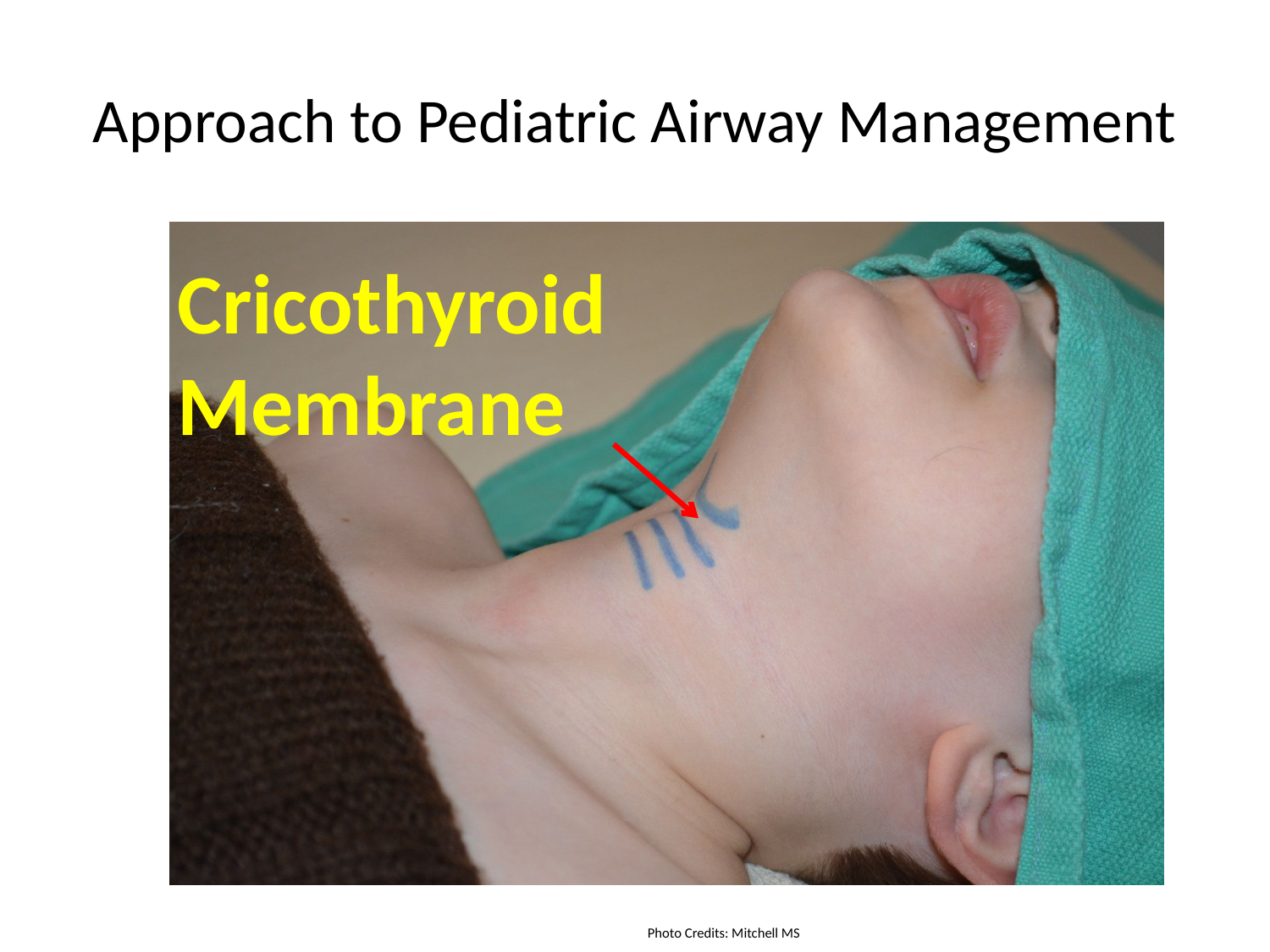

# Approach to Pediatric Airway Management
Cricothyroid Membrane
Photo Credits: Mitchell MS

## Slide 10
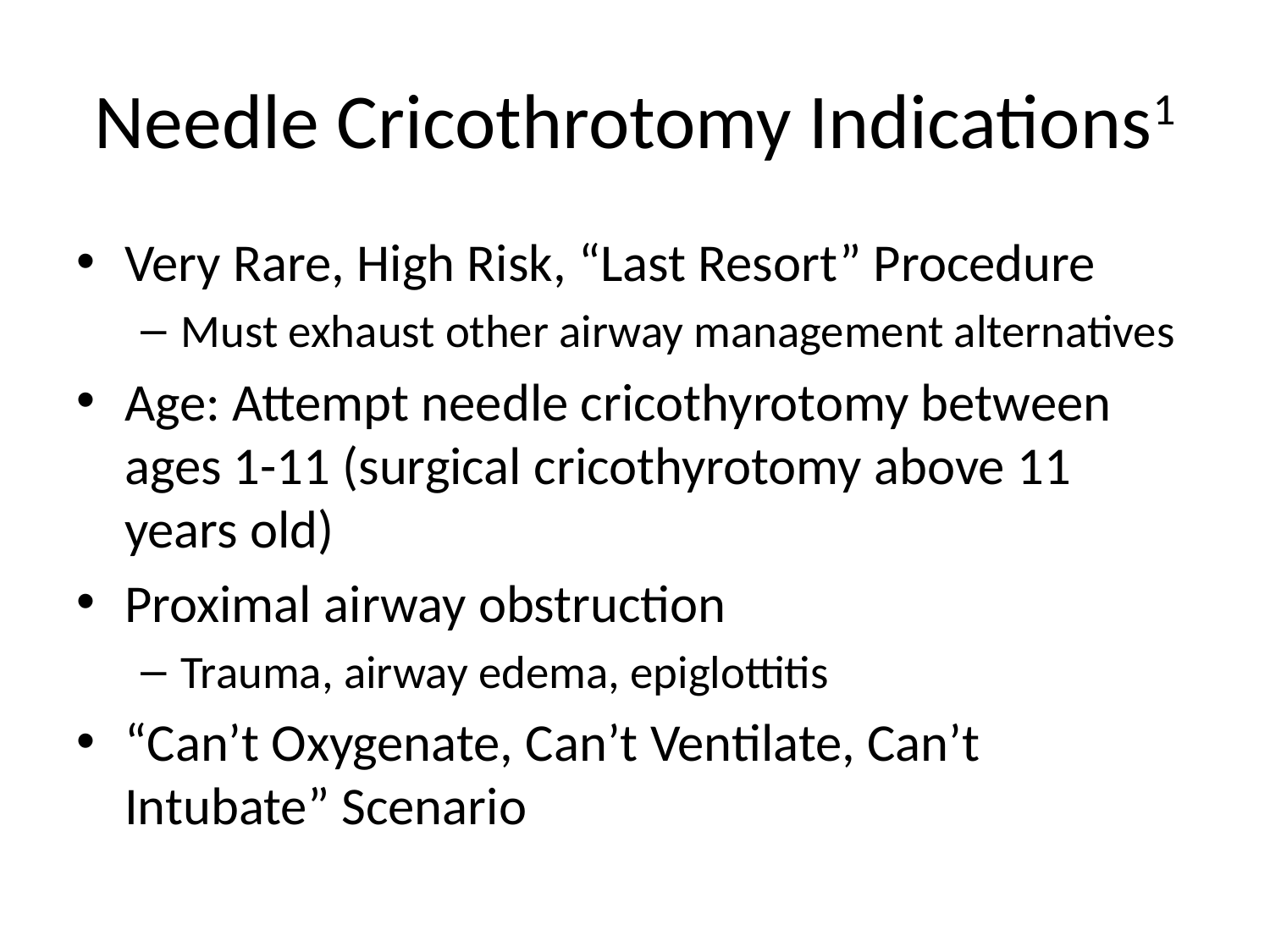

# Needle Cricothrotomy Indications1
Very Rare, High Risk, “Last Resort” Procedure
Must exhaust other airway management alternatives
Age: Attempt needle cricothyrotomy between ages 1-11 (surgical cricothyrotomy above 11 years old)
Proximal airway obstruction
Trauma, airway edema, epiglottitis
“Can’t Oxygenate, Can’t Ventilate, Can’t Intubate” Scenario

## Slide 11
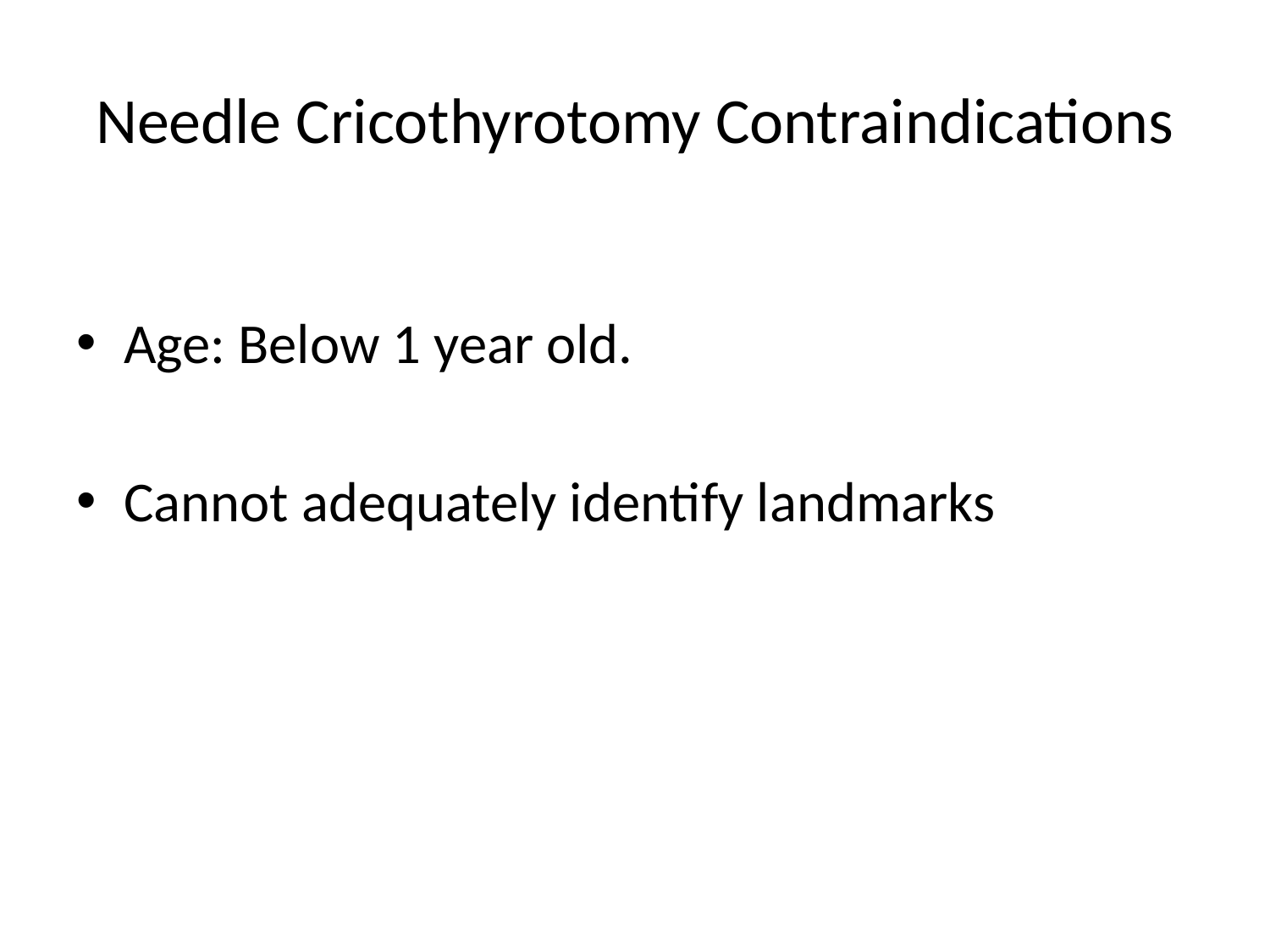

# Needle Cricothyrotomy Contraindications
Age: Below 1 year old.
Cannot adequately identify landmarks

## Slide 12
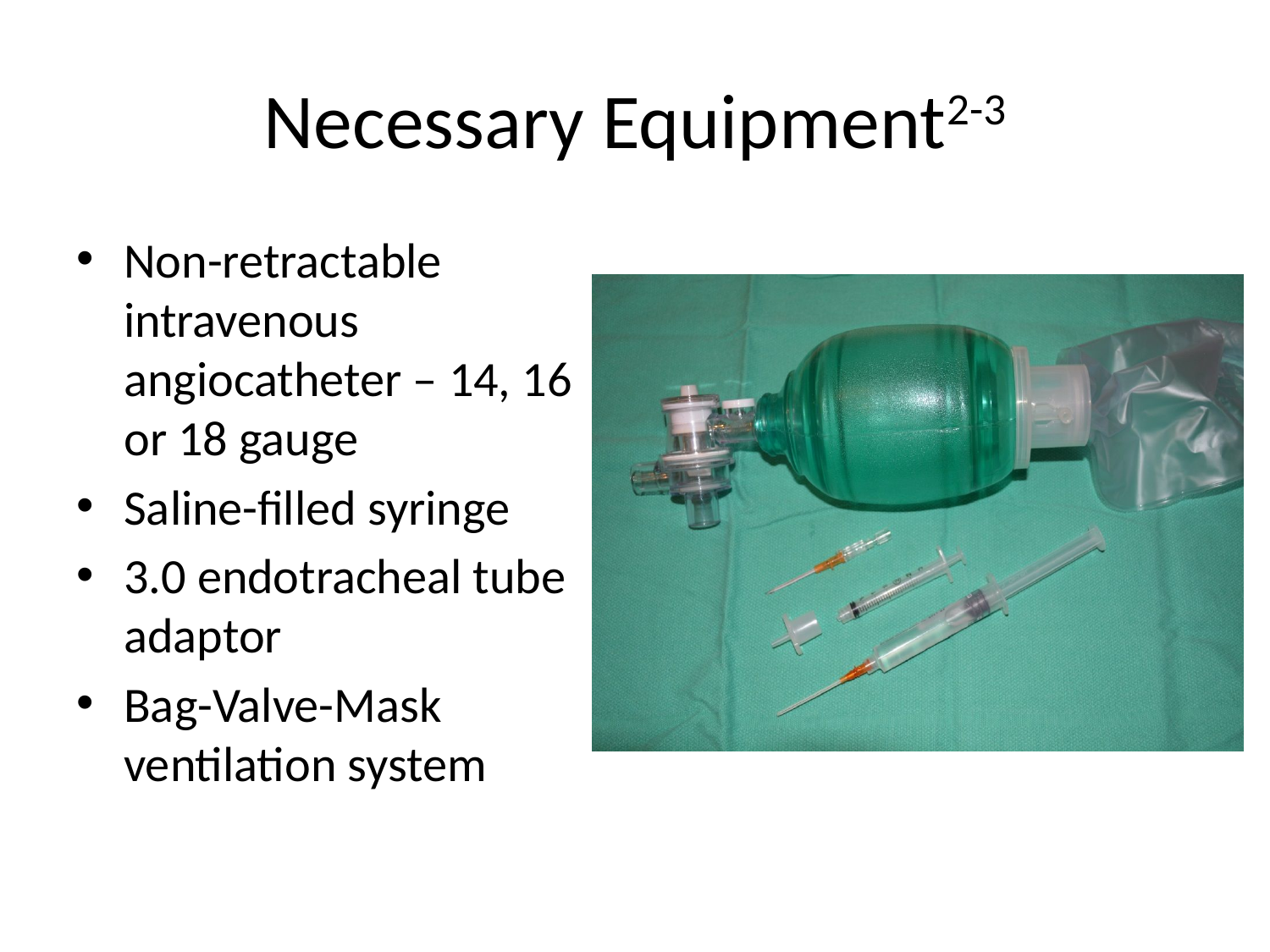

# Necessary Equipment2-3
Non-retractable intravenous angiocatheter – 14, 16 or 18 gauge
Saline-filled syringe
3.0 endotracheal tube adaptor
Bag-Valve-Mask ventilation system

## Slide 13
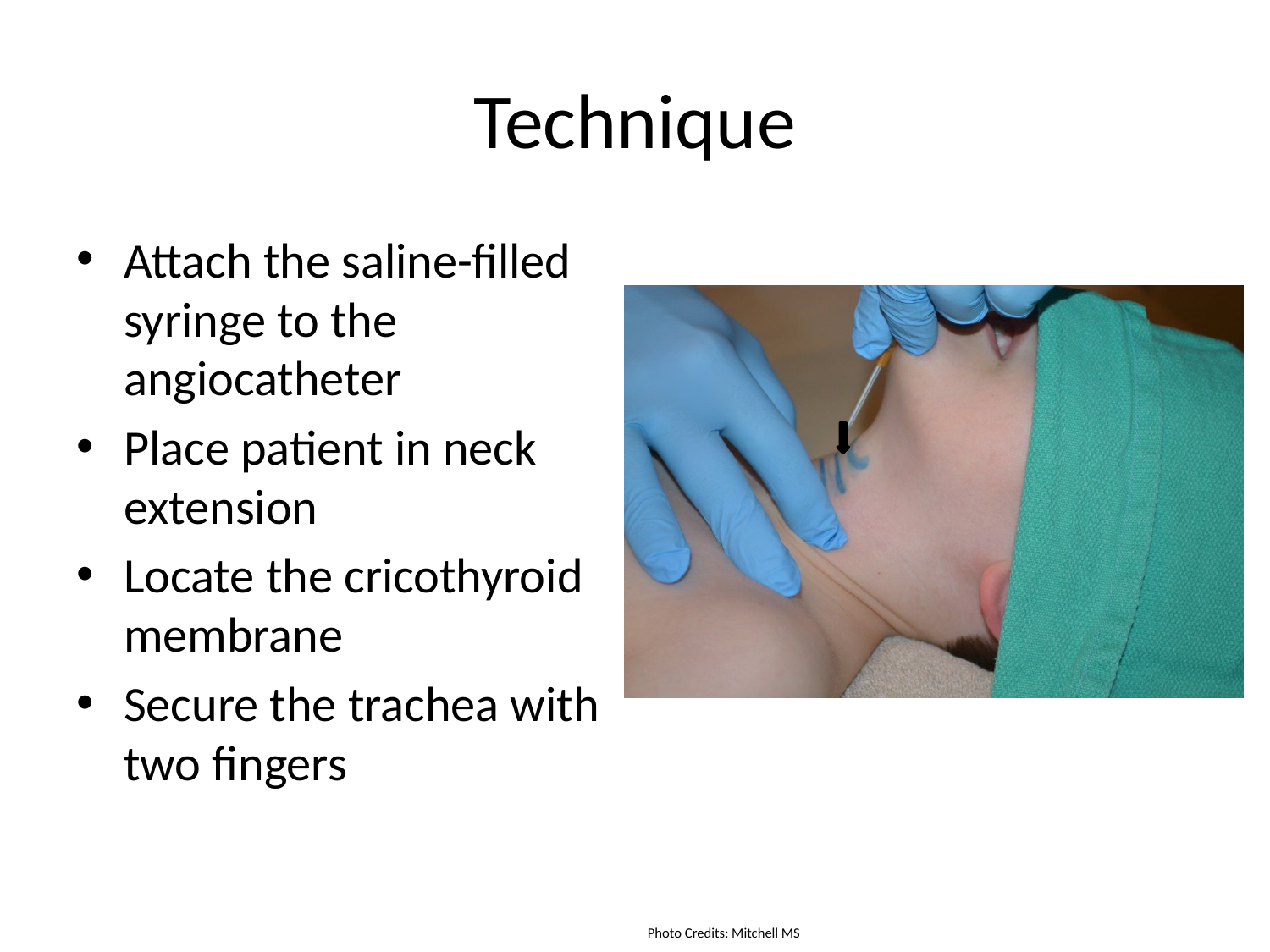

# Technique
Attach the saline-filled syringe to the angiocatheter
Place patient in neck extension
Locate the cricothyroid membrane
Secure the trachea with two fingers
Photo Credits: Mitchell MS

## Slide 14
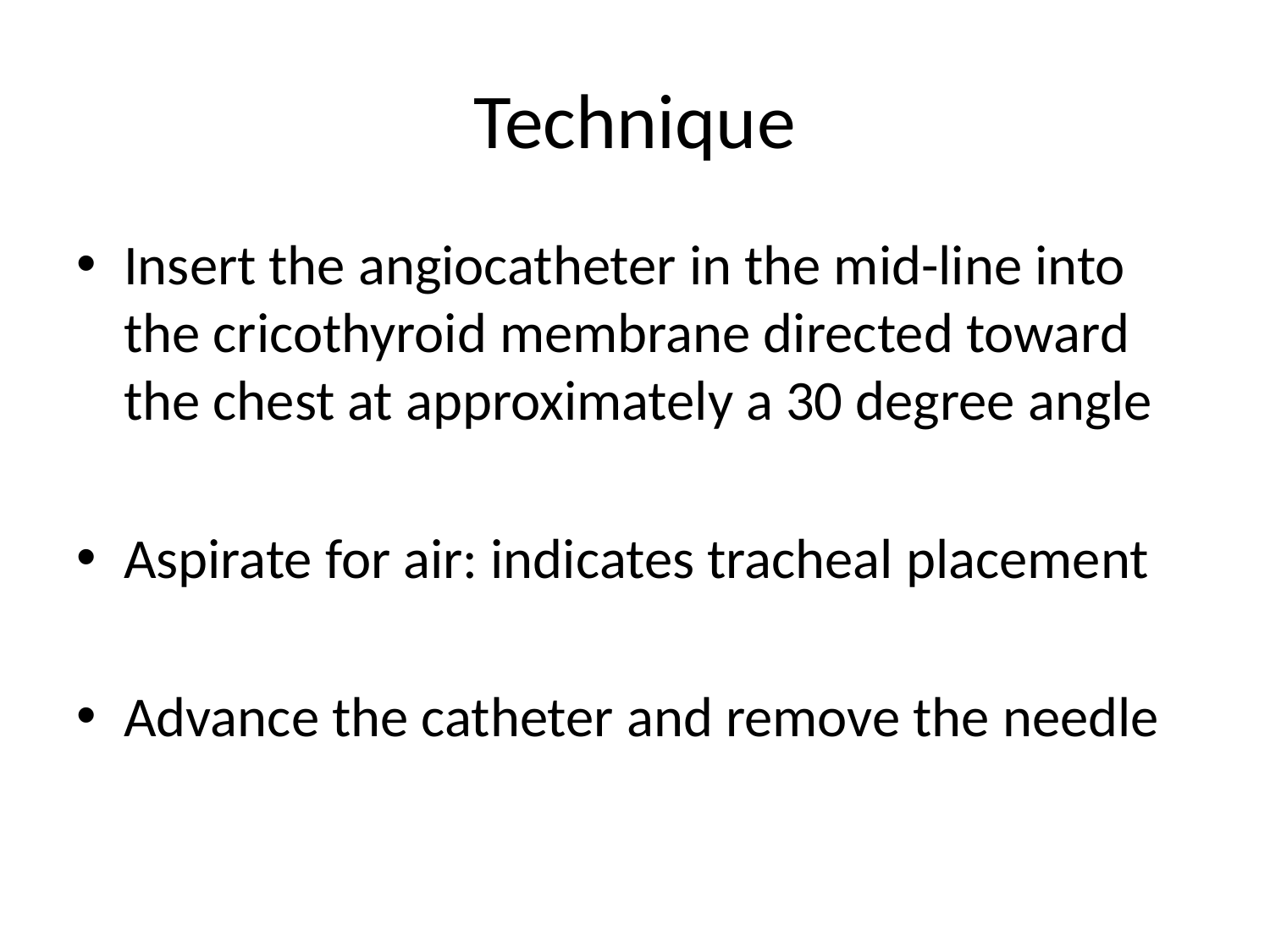

# Technique
Insert the angiocatheter in the mid-line into the cricothyroid membrane directed toward the chest at approximately a 30 degree angle
Aspirate for air: indicates tracheal placement
Advance the catheter and remove the needle

## Slide 15
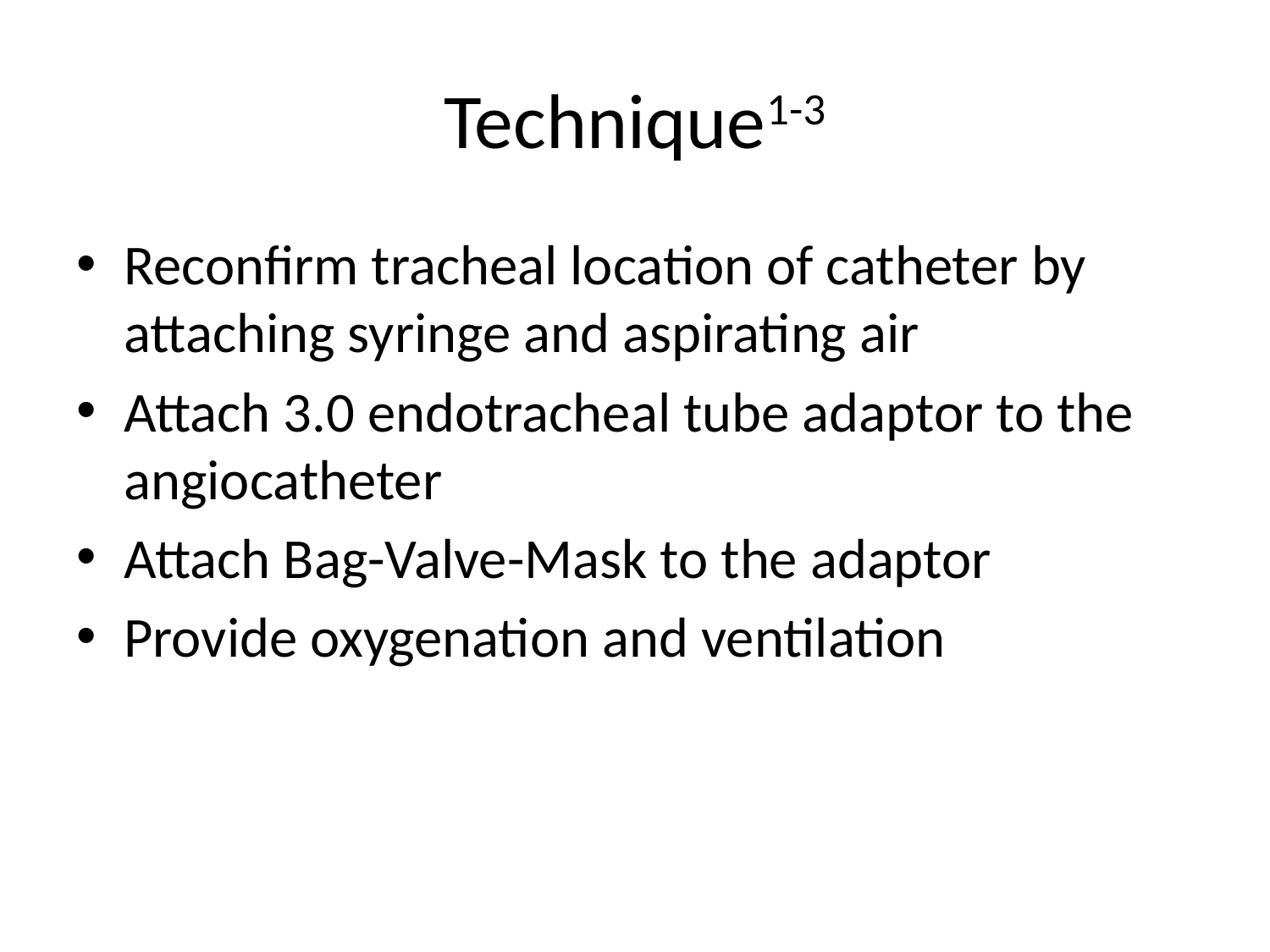

# Technique1-3
Reconfirm tracheal location of catheter by attaching syringe and aspirating air
Attach 3.0 endotracheal tube adaptor to the angiocatheter
Attach Bag-Valve-Mask to the adaptor
Provide oxygenation and ventilation

## Slide 16
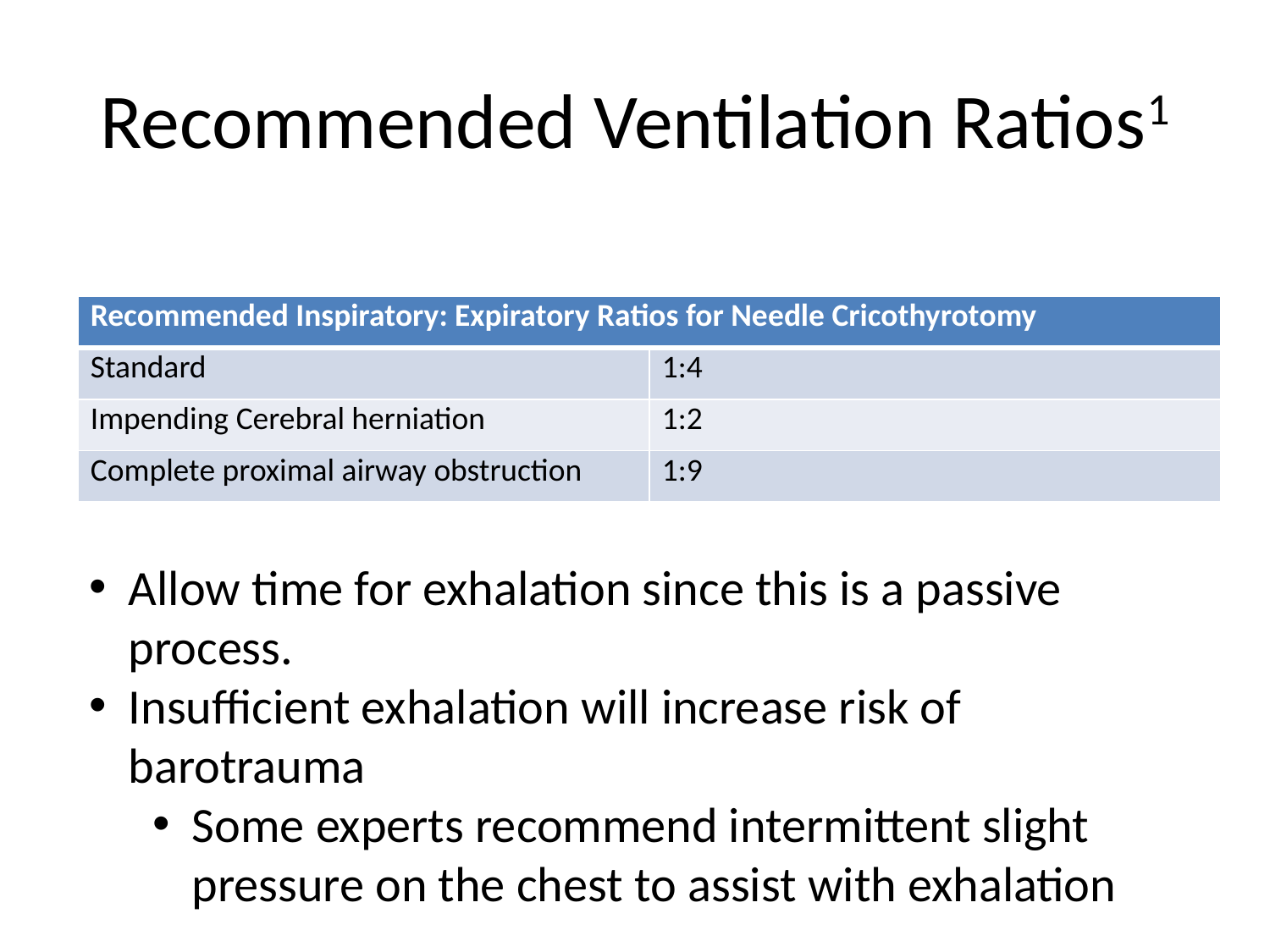

# Recommended Ventilation Ratios1
| Recommended Inspiratory: Expiratory Ratios for Needle Cricothyrotomy | |
| --- | --- |
| Standard | 1:4 |
| Impending Cerebral herniation | 1:2 |
| Complete proximal airway obstruction | 1:9 |
Allow time for exhalation since this is a passive process.
Insufficient exhalation will increase risk of barotrauma
Some experts recommend intermittent slight pressure on the chest to assist with exhalation

## Slide 17
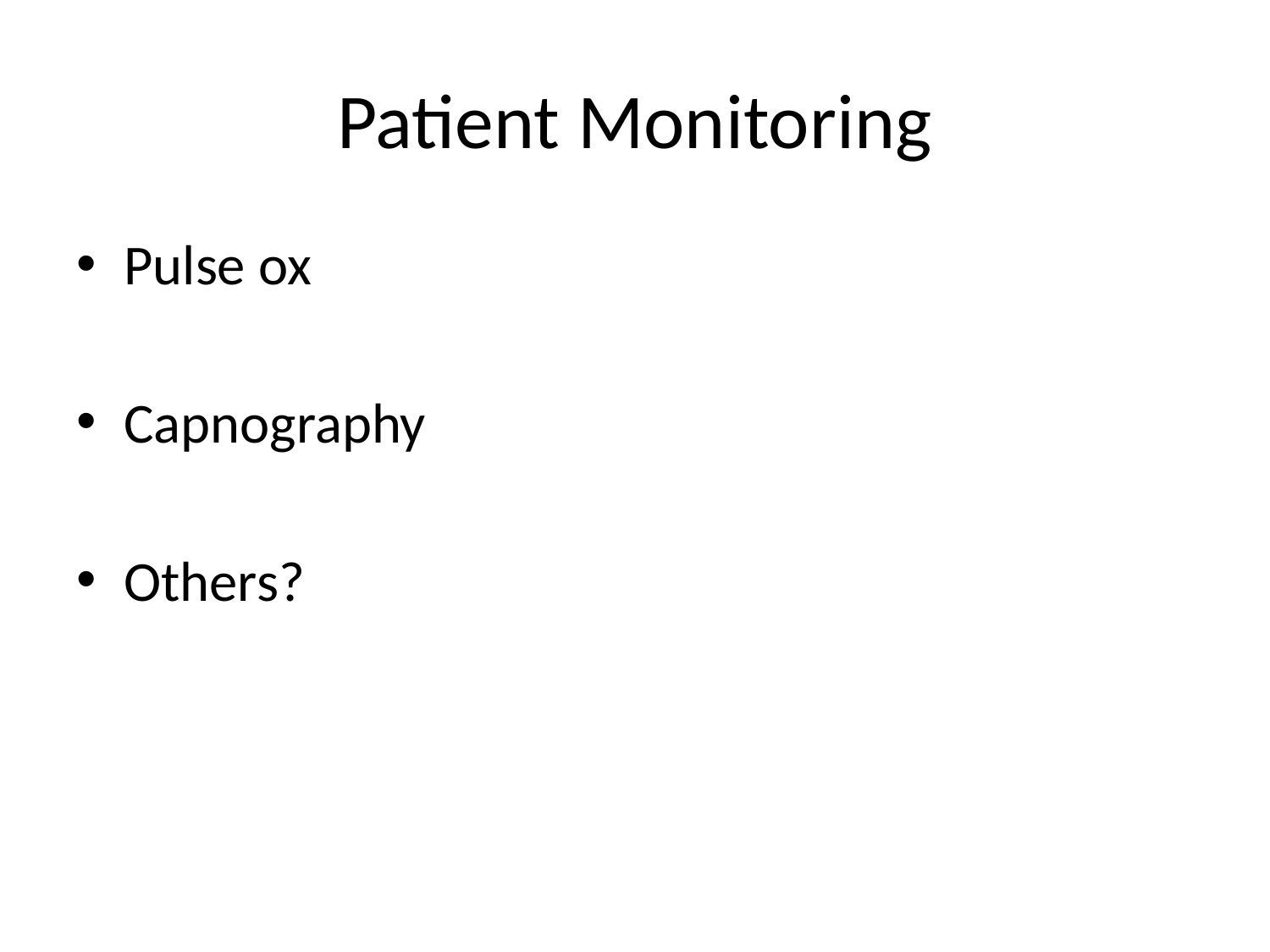

# Patient Monitoring
Pulse ox
Capnography
Others?

## Slide 18
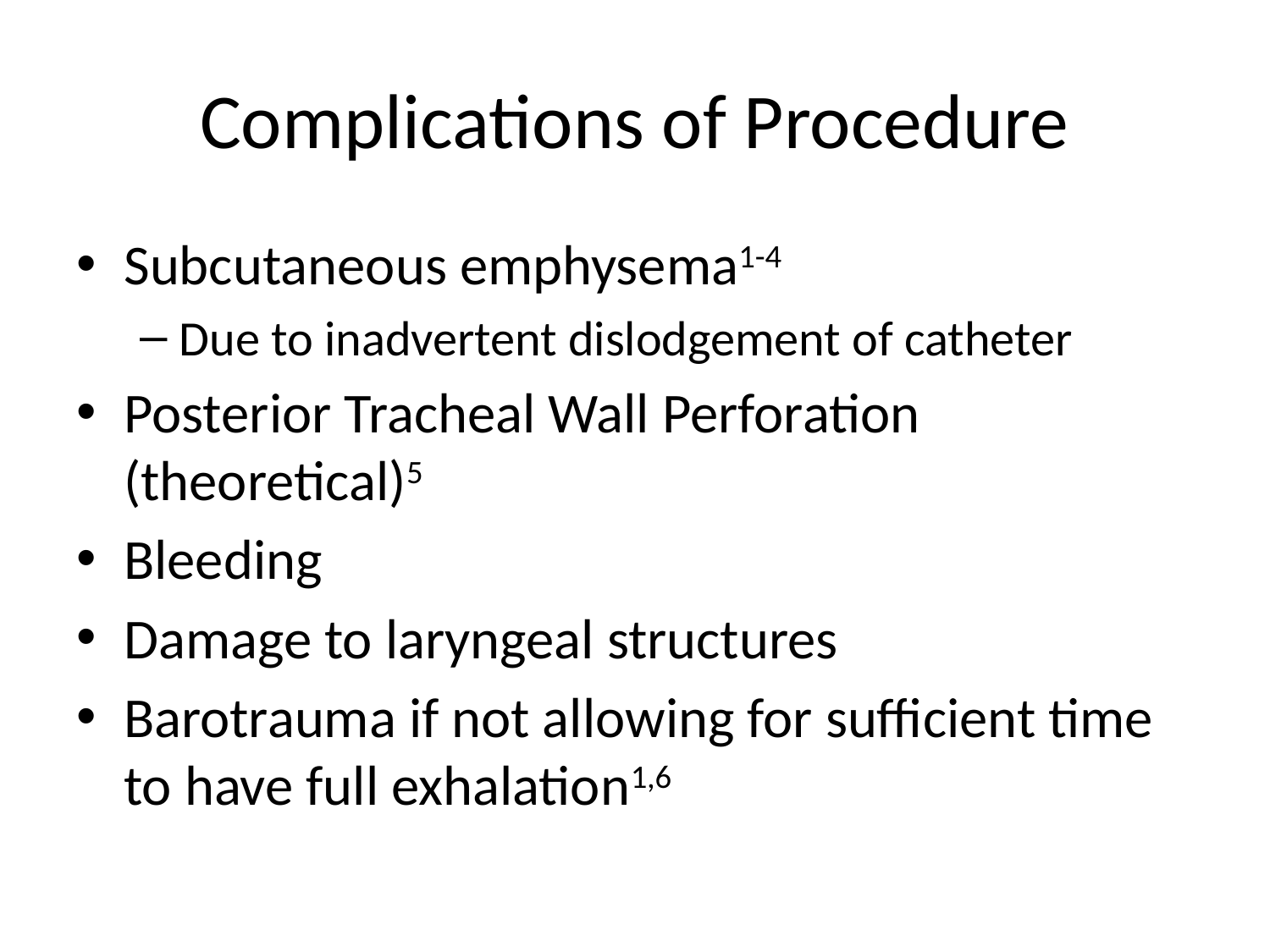

# Complications of Procedure
Subcutaneous emphysema1-4
Due to inadvertent dislodgement of catheter
Posterior Tracheal Wall Perforation (theoretical)5
Bleeding
Damage to laryngeal structures
Barotrauma if not allowing for sufficient time to have full exhalation1,6

## Slide 19
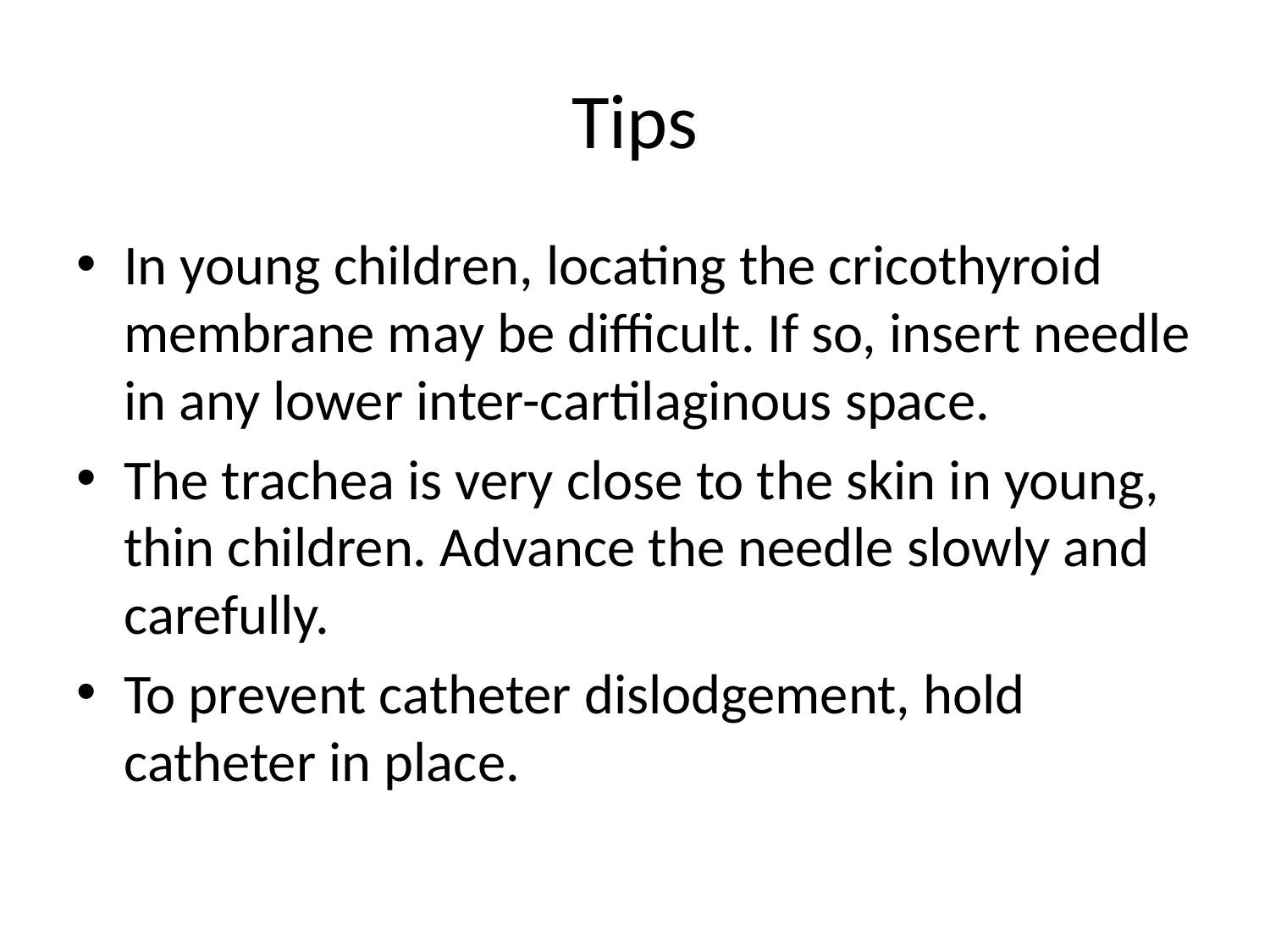

# Tips
In young children, locating the cricothyroid membrane may be difficult. If so, insert needle in any lower inter-cartilaginous space.
The trachea is very close to the skin in young, thin children. Advance the needle slowly and carefully.
To prevent catheter dislodgement, hold catheter in place.

## Slide 20
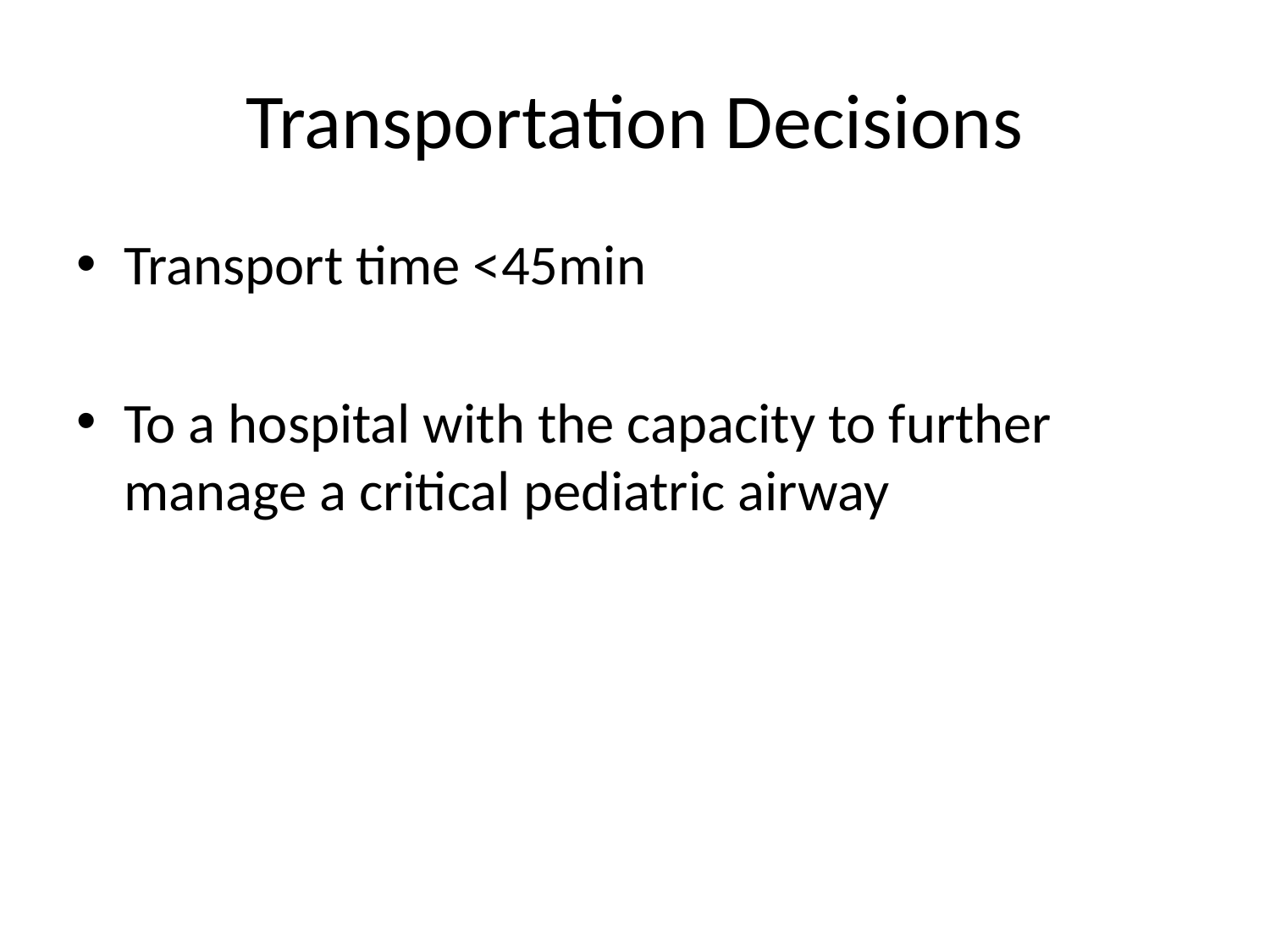

# Transportation Decisions
Transport time <45min
To a hospital with the capacity to further manage a critical pediatric airway

## Slide 21
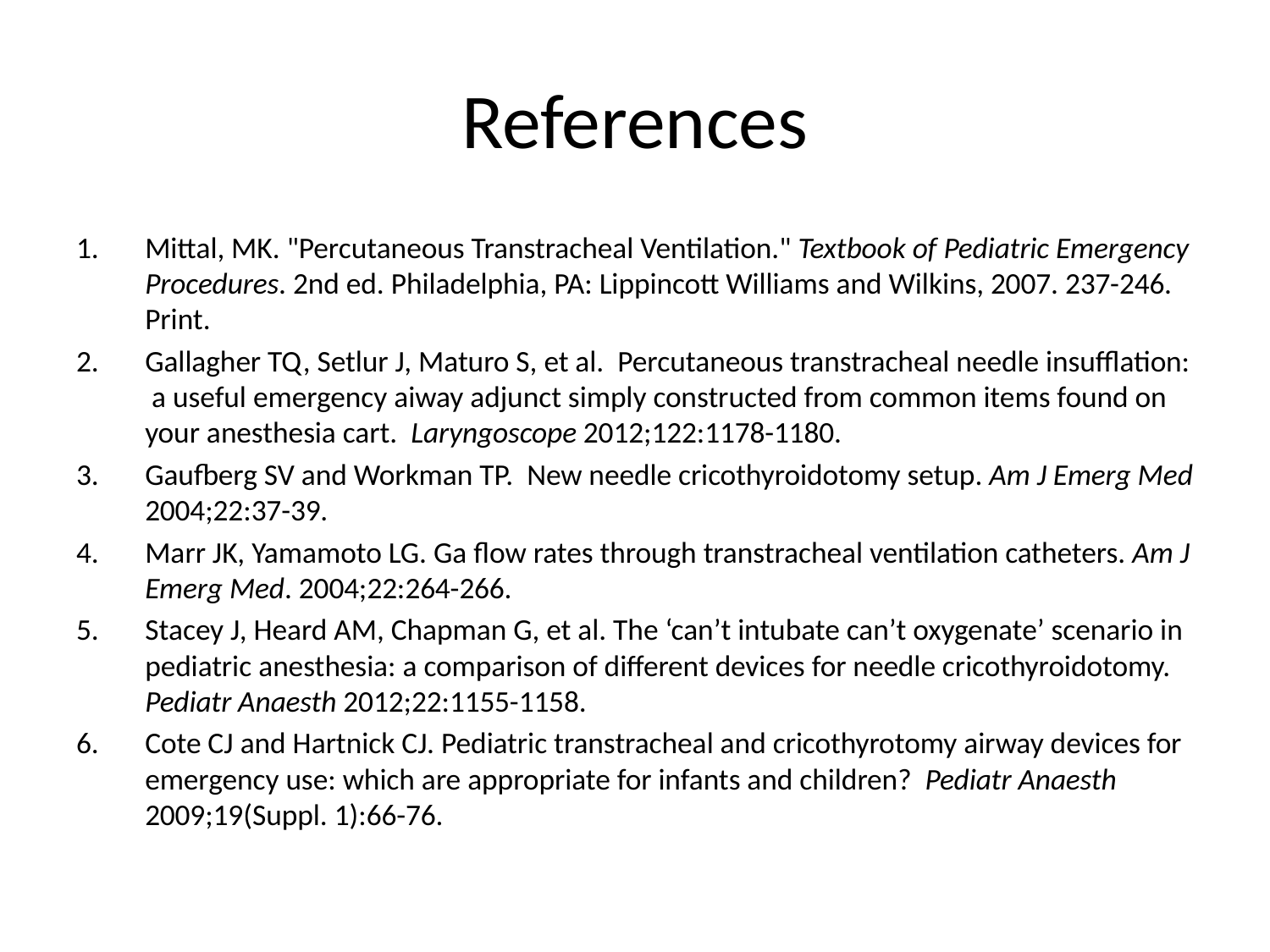

# References
Mittal, MK. "Percutaneous Transtracheal Ventilation." Textbook of Pediatric Emergency Procedures. 2nd ed. Philadelphia, PA: Lippincott Williams and Wilkins, 2007. 237-246. Print.
Gallagher TQ, Setlur J, Maturo S, et al. Percutaneous transtracheal needle insufflation: a useful emergency aiway adjunct simply constructed from common items found on your anesthesia cart. Laryngoscope 2012;122:1178-1180.
Gaufberg SV and Workman TP. New needle cricothyroidotomy setup. Am J Emerg Med 2004;22:37-39.
Marr JK, Yamamoto LG. Ga flow rates through transtracheal ventilation catheters. Am J Emerg Med. 2004;22:264-266.
Stacey J, Heard AM, Chapman G, et al. The ‘can’t intubate can’t oxygenate’ scenario in pediatric anesthesia: a comparison of different devices for needle cricothyroidotomy. Pediatr Anaesth 2012;22:1155-1158.
Cote CJ and Hartnick CJ. Pediatric transtracheal and cricothyrotomy airway devices for emergency use: which are appropriate for infants and children? Pediatr Anaesth 2009;19(Suppl. 1):66-76.
