# Supplementary material for: Pediatric Needle Cricothyrotomy: A Case for Simulation in Prehospital Medicine
Source: MedEdPORTAL. 2017 Jun 2;13:10589. doi: 10.15766/mep_2374-8265.10589 (PMC6338176; doi:10.15766/mep_2374-8265.10589)
Supplement: Supplementary file 1 — A. Simulation Case.docx B. PowerPoint Presentation.pptx C. Participant Evaluation Tool.docx D. Pre- and Posttest.docx E. Fetal Pig Model.docx F. Hardware Store Model.docx G. Correct Procedure Technique Explained.docx H. Needle Kit Image.JPG I. Angioedema Image.JPG J. Urticaria Image.jpg [file mep-13-10589-s001.zip › C. Participant Evaluation Tool.docx]

**Participant Evaluation Tool: Pediatric Anaphylaxis with complete airway obstruction**

**NAME:___________________________________________________**

| **SCENE SIZE-­‐UP** | **Weight** | **Awarded** | **Notes** |
| --- | --- | --- | --- |
| Determines scene is safe*** | 2 |  |  |
| Determines # patients, nature of illness & severity | 1 |  |  |
| Requests additional resources if needed | 1 |  |  |
| Uses appropriate personal protective equipment | 1 |  |  |
| **PRIMARY SURVEY / RESUSCITATION** |  |  |  |
| AVPU | 2 |  |  |
| Determines CC / Apparent life-­‐threats | 2 |  |  |
| **ABC** |  |  |  |
| Assessment of airway*** | 4 |  |  |
| Assesses breathing*** | 4 |  |  |
| Assures adequate ventilation*** | 4 |  |  |
| Oxygen therapy | 2 |  |  |
| Checks pulse*** | 4 |  |  |
| Assesses skin (color, temperature or condition) | 2 |  |  |
| Conserves body heat | 2 |  |  |
| Identifies priority, treatment/transport decision | 4 |  |  |
| **HISTORY TAKING: History of present illness** |  |  |  |
| Onset | 2 |  |  |
| Provocation | 2 |  |  |
| Quality | 2 |  |  |
| Radiation | 2 |  |  |
| Severity | 2 |  |  |
| Time | 2 |  |  |
| Clarifying questions of associated S/S | 2 |  |  |
| **PAST MEDICAL HISTORY** |  |  |  |
| Allergies | 2 |  |  |
| Medications | 2 |  |  |
| PMH | 2 |  |  |
| Last oral intake | 2 |  |  |
| Events leading to present illness / injury | 2 |  |  |
| SECONDARY ASSESSMENT |  |  |  |
| General | 1 |  |  |
| Head, mouth, nose, face, scalp and ears | 1 |  |  |
| Neck | 1 |  |  |
| Chest, inspects, palpates, auscultates | 1 |  |  |
| Cardiovascular | 1 |  |  |
| Abdomen, inspects, palpates | 1 |  |  |
| Neurological | 1 |  |  |
| Skin | 1 |  |  |
| Musculoskeletal | 1 |  |  |
| GU | 1 |  |  |

| **VITAL SIGNS** |  |  |  |
| --- | --- | --- | --- |
| BP | 2 |  |  |
| Pulse (HR)*** | 2 |  |  |
| Respiratory rate / quality*** | 2 |  |  |
| SaO2 | 2 |  |  |
| FSBS | 2 |  |  |
| EtCO2 | 1 |  |  |
| **INTERVENTION** |  |  |  |
| Immediate IM Epi 1:1000 | 5 |  |  |
| Repeat IM Epi 1:1000 | 5 |  |  |
| Intraosseous started | 5 |  |  |
| Benadryl IO/IM | 2 |  |  |
| Corticosteroid IO/IM | 4 |  |  |
| Nebulized Epi/Albuterol | 4 |  |  |
| Bag Valve Mask Ventilation | 4 |  |  |
| Determines “Can’t ventilate, Can’t oxygenate” | 2 |  |  |
| Needle Cricothyrotomy | 10 |  |  |
| Fluid Bolus | 4 |  |  |
| Considers/Administers Epi Drip Correctly | 5 |  |  |
| **REASSESSMENT** |  |  |  |
| Determines when / how to reassess changes in  condition | 2 |  |  |
| **Repeat VITAL SIGNS** |  |  |  |
| BP | 2 |  |  |
| Pulse (HR) | 2 |  |  |
| Respiratory rate / quality | 2 |  |  |
| SaO2 | 2 |  |  |
| EtCO2 (if respiratory complaint) | 1 |  |  |
| Items in RED*** not completed | -5 each |  |  |
| Action that caused SERIOUS HARM 🡪 Critical FAIL | XXX |  |  |
| SCENARIO TOTAL SCORE |  |  | PASS FAIL |

Provider: Date:

Evaluator:
