# Supplementary material for: Pediatric Needle Cricothyrotomy: A Case for Simulation in Prehospital Medicine
Source: MedEdPORTAL. 2017 Jun 2;13:10589. doi: 10.15766/mep_2374-8265.10589 (PMC6338176; doi:10.15766/mep_2374-8265.10589)
Supplement: Supplementary file 1 — A. Simulation Case.docx B. PowerPoint Presentation.pptx C. Participant Evaluation Tool.docx D. Pre- and Posttest.docx E. Fetal Pig Model.docx F. Hardware Store Model.docx G. Correct Procedure Technique Explained.docx H. Needle Kit Image.JPG I. Angioedema Image.JPG J. Urticaria Image.jpg [file mep-13-10589-s001.zip › D. Pre- and Posttest.docx]

**Advanced Pediatric Airway - Cricothyrotomy**

*Pre-Training Survey*

1. **I feel comfortable with my skills to successfully manage airway emergencies in young children.**

1 2 3 4 5

Strongly Disagree Disagree Neutral Agree Strongly Agree

1. **I am confident in my technical ability to perform a needle cricothyrotomy when necessary.**

1 2 3 4 5

Strongly Disagree Disagree Neutral Agree Strongly Agree

1. **I know all of the equipment that I will need to have available when performing a needle cricothyrotomy and successfully ventilating after the procedure.**

1 2 3 4 5

Strongly Disagree Disagree Neutral Agree Strongly Agree

1. **List one indication to perform a needle cricothyrotomy:**

______________________________

1. **List one contraindication to performing a needle cricothyrotomy:**

______________________________

1. **What is the recommended standard inspiration to expiration ratio with a needle cricothyrotomy?**
   1. 1:1
   2. 1:2
   3. 1:4
   4. 1:10

**Advanced Pediatric Airway - Cricothyrotomy**

*Post-Training Survey*

1. **I feel comfortable with my skills to successfully manage airway emergencies in young children.**

1 2 3 4 5

Strongly Disagree Disagree Neutral Agree Strongly Agree

1. **I am confident in my technical ability to perform a needle cricothyrotomy when necessary.**

1 2 3 4 5

Strongly Disagree Disagree Neutral Agree Strongly Agree

1. **I know all of the equipment that I will need to have available when performing a needle cricothyrotomy and successfully ventilating after the procedure.**

1 2 3 4 5

Strongly Disagree Disagree Neutral Agree Strongly Agree

1. **List one indication to perform a needle cricothyrotomy:**

______________________________

1. **List one contraindication to performing a needle cricothyrotomy:**

______________________________

1. **What is the recommended standard inspiration to expiration ratio with a needle cricothyrotomy?**
   1. 1:1
   2. 1:2
   3. 1:4
   4. 1:10
2. **This training exercise was valuable to enhance my expertise with needle cricothyrotomy.**

1 2 3 4 5

Strongly Disagree Disagree Neutral Agree Strongly Agree

1. **The simulation model used today provided appropriate realism to learn this procedure.**

1 2 3 4 5

Strongly Disagree Disagree Neutral Agree Strongly Agree
