# Supplementary material for: Pediatric Needle Cricothyrotomy: A Case for Simulation in Prehospital Medicine
Source: MedEdPORTAL. 2017 Jun 2;13:10589. doi: 10.15766/mep_2374-8265.10589 (PMC6338176; doi:10.15766/mep_2374-8265.10589)
Supplement: Supplementary file 1 — A. Simulation Case.docx B. PowerPoint Presentation.pptx C. Participant Evaluation Tool.docx D. Pre- and Posttest.docx E. Fetal Pig Model.docx F. Hardware Store Model.docx G. Correct Procedure Technique Explained.docx H. Needle Kit Image.JPG I. Angioedema Image.JPG J. Urticaria Image.jpg [file mep-13-10589-s001.zip › E. Fetal Pig Model.docx]

Appendix B - Fetal Pig Model

The 14” non-injected fetal pig is prepped by making an incision through the neck skin. Then simple and rapid blunt dissection with allow the airways structures to be easily palpated. The authors found that the airway is normally filled with clear fluid. It is beneficial to place a needle in the trachea (near the thoracic inlet) and insufflate the airway with 10-20cc of air. The pig is now ready to be used as a model for pediatric needle cricothyrotomy.
