# Supplementary material for: Pediatric Needle Cricothyrotomy: A Case for Simulation in Prehospital Medicine
Source: MedEdPORTAL. 2017 Jun 2;13:10589. doi: 10.15766/mep_2374-8265.10589 (PMC6338176; doi:10.15766/mep_2374-8265.10589)
Supplement: Supplementary file 1 — A. Simulation Case.docx B. PowerPoint Presentation.pptx C. Participant Evaluation Tool.docx D. Pre- and Posttest.docx E. Fetal Pig Model.docx F. Hardware Store Model.docx G. Correct Procedure Technique Explained.docx H. Needle Kit Image.JPG I. Angioedema Image.JPG J. Urticaria Image.jpg [file mep-13-10589-s001.zip › F. Hardware Store Model.docx]

Appendix C - “Hardware Store” model


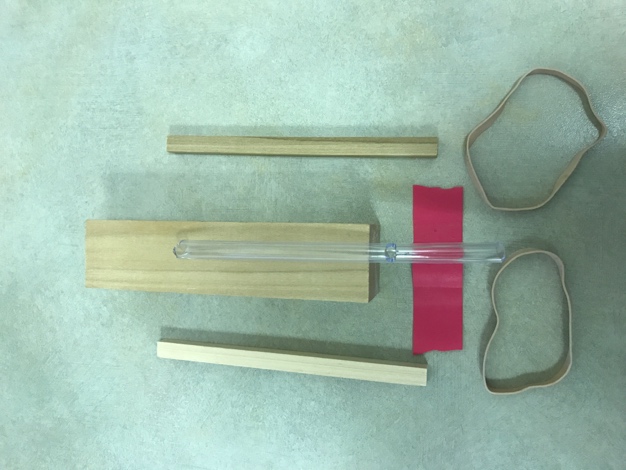


Parts List:

1. 1”x1”x5” block (poplar wood used here)
2. ¼” square dowel x 5” (2)
3. 3/8” inner diameter tubing x 5”
4. Rubber bands (2)
5. Non-latex tourniquet 3”


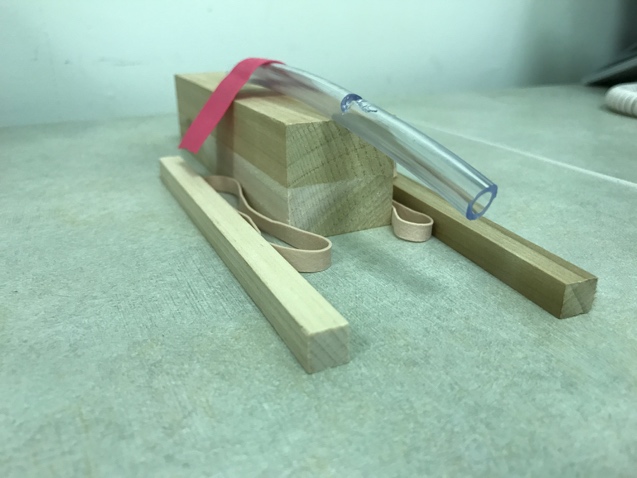


Prep: Cut a notch out of the tubing closer to one end than the other. This notch is the simulated cricothyroid space.


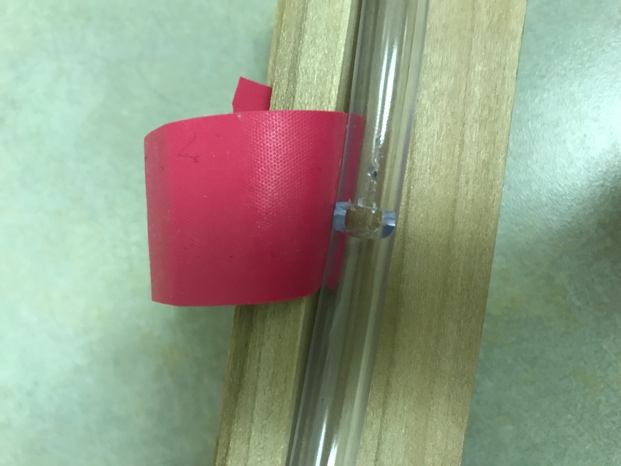

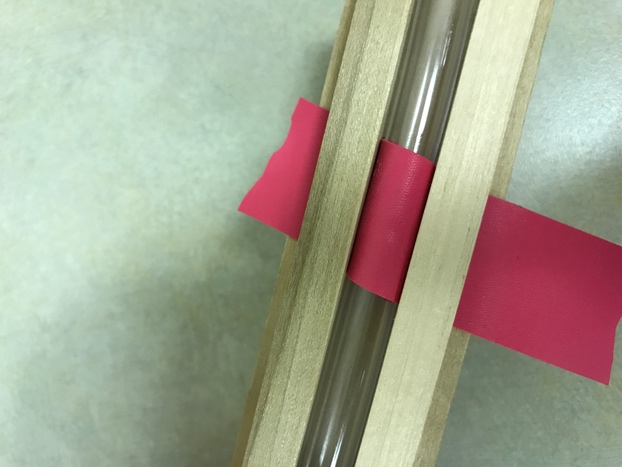


The non-latex tourniquet is placed under the square dowel, over the tubing and under the second square dowel before rubber bands are placed on each end of the model. To reset the model a new portion or piece of tourniquet is used.

Procedure


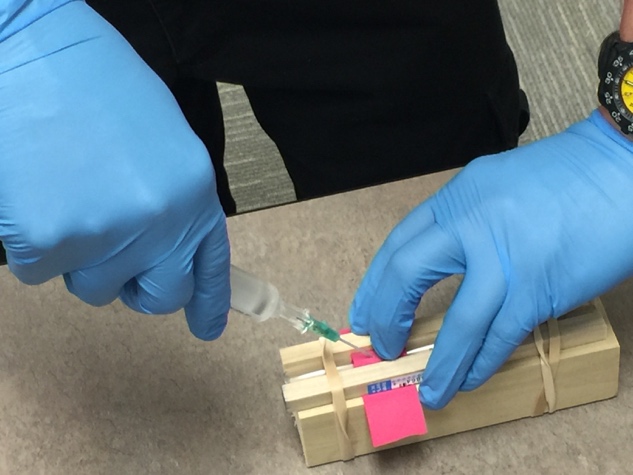


Technique of entering the cricothyroid membrane.


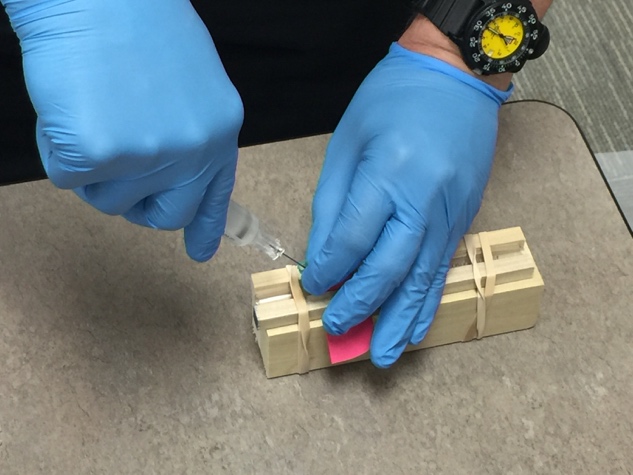


Advancing the catheter.


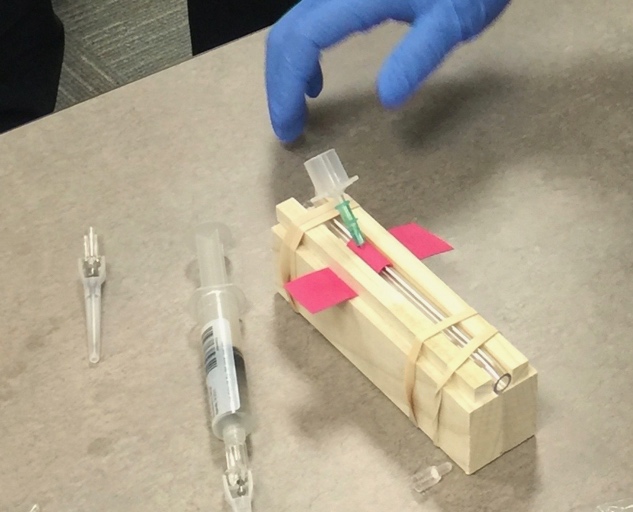


Attach 3.0 ET tube adapter. Be certain to stress the importance of constantly holding the catheter in place.
