# Supplementary material for: Pediatric Needle Cricothyrotomy: A Case for Simulation in Prehospital Medicine
Source: MedEdPORTAL. 2017 Jun 2;13:10589. doi: 10.15766/mep_2374-8265.10589 (PMC6338176; doi:10.15766/mep_2374-8265.10589)
Supplement: Supplementary file 1 — A. Simulation Case.docx B. PowerPoint Presentation.pptx C. Participant Evaluation Tool.docx D. Pre- and Posttest.docx E. Fetal Pig Model.docx F. Hardware Store Model.docx G. Correct Procedure Technique Explained.docx H. Needle Kit Image.JPG I. Angioedema Image.JPG J. Urticaria Image.jpg [file mep-13-10589-s001.zip › G. Correct Procedure Technique Explained.docx]

Appendix G

Airway Management - The participant should recognize the patient’s airway compromise indicated by difficulty in providing ventilation and oxygenation. The patient should be optimally positioned to open the airway. Oxygenation should be attempted with the appropriately sized face mask and then escalate to bag valve mask. The participant should utilize nasal pharyngeal airways to help oxygenate and ventilate the patient. An attempt to visualize any foreign objects in the airway should be made. The participant should recognize that initial attempts to oxygenate and ventilate have failed and should pursue either blind insertion airway or direct laryngoscopy. After all available primary equipment to oxygenate and ventilate the patient have failed, the participant should recognize the need to perform needle cricothyrotomy.

Needle Cricothyrotomy – The participant should evaluate the patient for possible needle cricothyrotomy and evaluate neck anatomy for any possible difficulties. Gather the supplies needed for appropriate placement of needle cricothyrotomy. The participant will need to obtain a large bore angiocath (18 ga or larger), syringe half-filled with sterile saline, size 3 endotracheal tube adapter, and an alcohol swab. The patient should be positioned with fully hyperextended neck and the participant should identify the cricoid cartilage. The patient’s neck should be swabbed with alcohol. The participant should hold the patient’s trachea in place with the non-dominant hand. The angiocath should be inserted at a 30-degree angle directed caudally into the cricoid cartilage while aspirating on the syringe. When air is aspirated, the catheter should be advanced while holding the needle in place. Next, the participant should attach the syringe directly to the catheter to ensure placement by aspirating air. When placement is confirmed, a 3.0 endotracheal tube adapter should be placed on the catheter. Then attach a bag valve mask to the endotracheal tube adaptor and assess for chest rise and fall. Appropriate airway positioning should be confirmed with end tidal carbon dioxide monitor and gradual improvement in the patient’s oxygen saturation. The participant should monitor for an improvement in vital signs while ventilating at the appropriate rate. The patient should be ventilated at an inspiration to expiration ratio of 1:4. Once the patient’s oxygenation improves, the participant should continue to hold the catheter in place because other means to secure this equipment is largely unsuccessful. Every effort should then be made to rapidly transport to a facility capable of establishing a definitive airway.
