# Supplementary figures and images for: Pediatric Needle Cricothyrotomy: A Case for Simulation in Prehospital Medicine
Source: MedEdPORTAL. 2017 Jun 2;13:10589. doi: 10.15766/mep_2374-8265.10589 (PMC6338176; doi:10.15766/mep_2374-8265.10589)

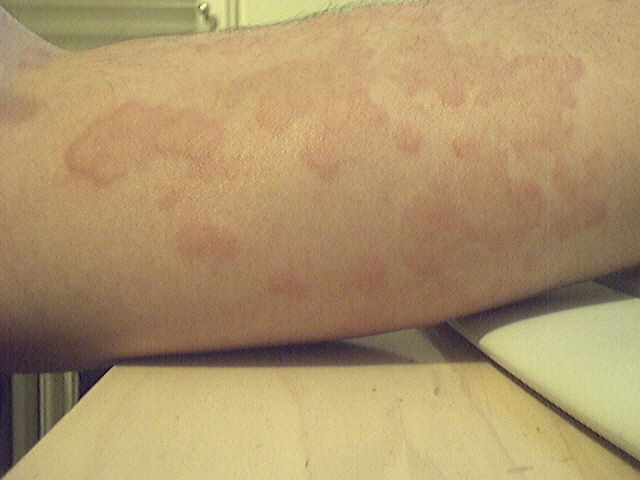

Supplement: Supplementary file 1 — A. Simulation Case.docx B. PowerPoint Presentation.pptx C. Participant Evaluation Tool.docx D. Pre- and Posttest.docx E. Fetal Pig Model.docx F. Hardware Store Model.docx G. Correct Procedure Technique Explained.docx H. Needle Kit Image.JPG I. Angioedema Image.JPG J. Urticaria Image.jpg [file mep-13-10589-s001.zip › J. Urticaria Image.jpg]

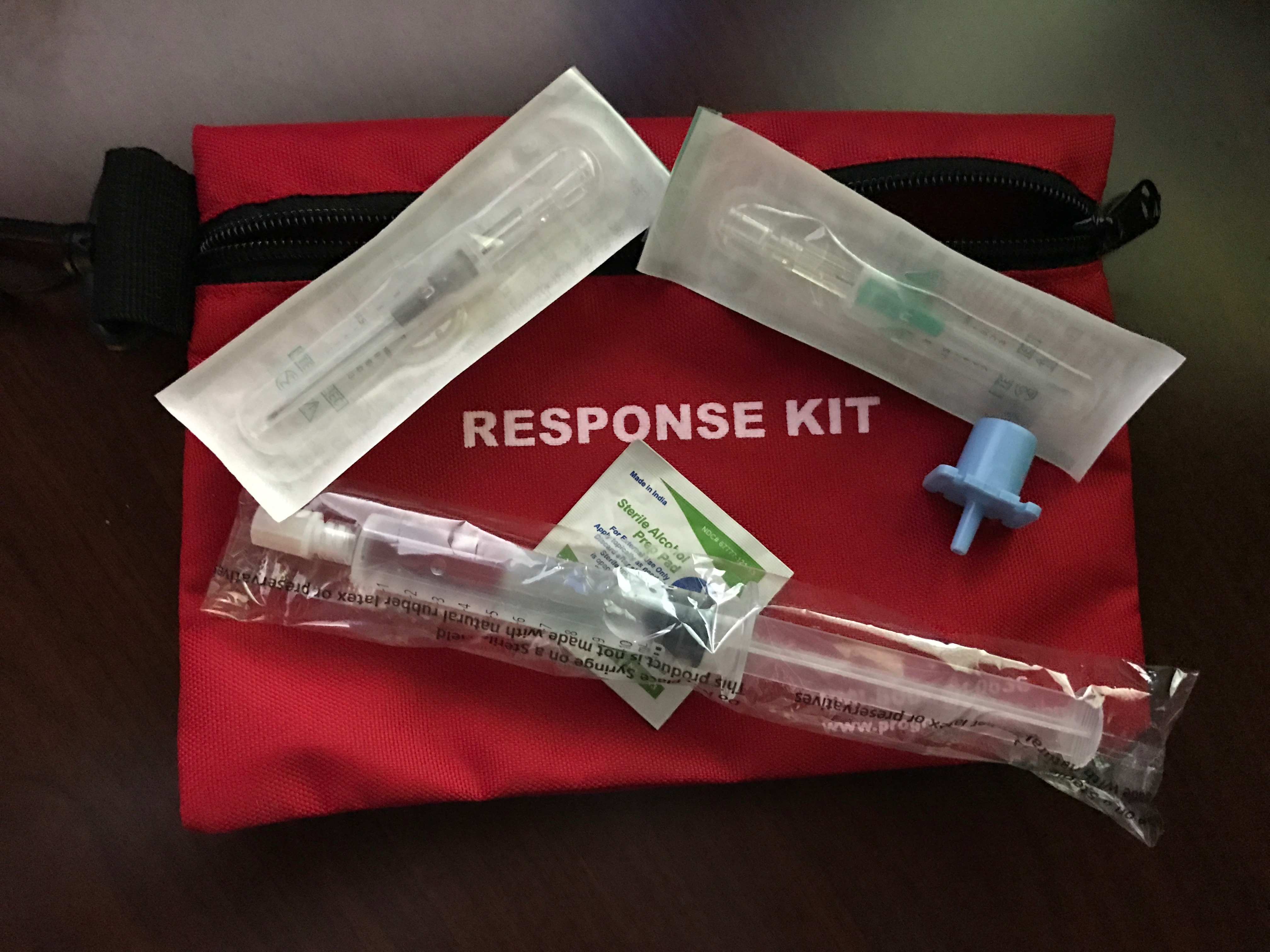

Supplement: Supplementary file 1 — A. Simulation Case.docx B. PowerPoint Presentation.pptx C. Participant Evaluation Tool.docx D. Pre- and Posttest.docx E. Fetal Pig Model.docx F. Hardware Store Model.docx G. Correct Procedure Technique Explained.docx H. Needle Kit Image.JPG I. Angioedema Image.JPG J. Urticaria Image.jpg [file mep-13-10589-s001.zip › H. Needle Kit Image.JPG]

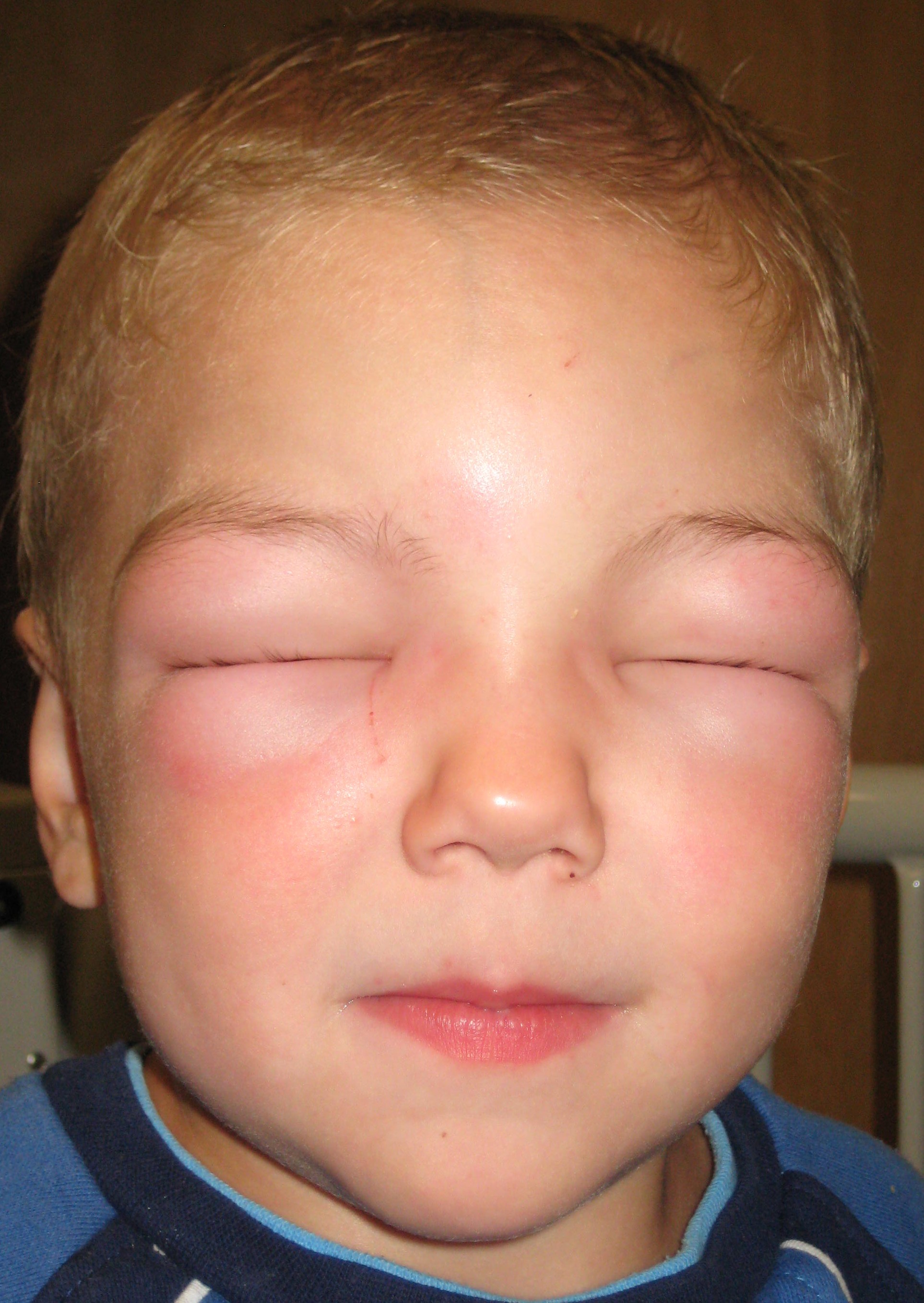

Supplement: Supplementary file 1 — A. Simulation Case.docx B. PowerPoint Presentation.pptx C. Participant Evaluation Tool.docx D. Pre- and Posttest.docx E. Fetal Pig Model.docx F. Hardware Store Model.docx G. Correct Procedure Technique Explained.docx H. Needle Kit Image.JPG I. Angioedema Image.JPG J. Urticaria Image.jpg [file mep-13-10589-s001.zip › I. Angioedema Image.JPG]
